# Spatially programmed polymorphic crystallization via microenvironment design

**DOI:** 10.1126/sciadv.aeg8728

**Published:** 2026-09-11

**Authors:** Zhenglian Qin, Yuchen Qiu, Jingyuan Zhang, Xiao Wei, Wen Wen, Tenglong Li, Huixue Su, Hanfei Gao, Jiangang Feng, Zhiyuan He, Yuchen Wu

**Affiliations:** ^1^ Laboratory of Bio-inspired Smart Interface Science, Technical Institute of Physics and Chemistry, Chinese Academy of Sciences, Beijing, 100190, China.; ^2^ College of Chemistry, Jilin University, Changchun 130012, China.; ^3^ State Key Laboratory of Bioinspired Interfacial Materials Science, Suzhou Institute for Advanced Research, University of Science and Technology of China, Suzhou, Jiangsu 215123, P. R. China.; ^4^ School of Future Technology, University of Chinese Academy of Sciences, Beijing, 101499, China.; ^5^ Department of Physics, Shanxi Datong University, Datong 037009, P.R. China.; ^6^ School of Materials Science and Engineering, Beijing Institute of Technology, Beijing, 100081, China.

## Abstract

Organic semiconductor crystals with long-range order and designable properties are pivotal in optoelectronic devices. Organic crystals bonded by weak intermolecular interactions usually follow non-classical crystallization trajectories, yielding polymorphs with distinctive properties but extremely difficult to control. We present a method for deterministic control of polymorphs, yielding organic microcrystal arrays with distinctive crystal structures and emission spectra. To elucidate the mechanisms of molecular self-assembly, we track the real-time dynamics of molecular nucleation, Ostwald’s ripening, and polymorph formation. Based on this method, we extended our regulation strategy to multiple molecules, achieving programmable polymorphic formation with broadly tunable emission spectra (450–705 nm) and anisotropic polarized emission. On a single substrate, we integrated multidimensional polymorphic information including position, wavelength, intensity, and polarization, constructing a high-density optical information storage platform with an information density of 2^15^ bits/cm^2^, thereby opening potential technological pathways for future optical information storage technologies.

## INTRODUCTION

Semiconductor crystals with long-range-ordered molecular packing, delocalized electrons and properties are pivotal in optoelectronic devices. Molecular packing in semiconductor crystals—intermolecular distances, relative orientations, and lattice symmetries—profoundly influence electronic band structures (*1*–*3*), charge carrier mobilities (*4*–*6*), and exciton diffusion lengths (*7*, *8*). For instance, coplanar versus herringbone π-π stacking geometries in organic semiconductors directly modulate orbital overlap, generating order-of-magnitude mobility variations and emission wavelength shifts exceeding 100 nm that govern the performance of solar cells, light-emitting diodes, and photodetectors (*9*, *10*). Such dramatic property tuning through different packing motifs of identical molecules—termed “polymorphs”—offers immense potential for programming material functionalities (*11*, *12*). Controllable polymorph selection would enable single-component systems to achieve distinctive properties: constructing emission-tunable pixel arrays, modulating charge transport channels, or creating spatially graded heterogeneous materials, thereby opening transformative design dimensions for self-assembled integrated devices.

Polymorph selection remains fundamentally unpredictable due to the intricate competition between thermodynamic stability and kinetic accessibility during nucleation, which is exquisitely sensitive to temperature (*13*, *14*), solvent (*15*), supersaturation (*16*), and interfacial chemistry (*17*). Controlling this outcome requires molecular-level understanding of how local crystallization environments modulate competing pathways and determine polymorph at the earliest nucleation stages (*18*, *19*). Unfortunately, direct experimental observation is hindered by the nanoscale spatial and millisecond temporal scales of nucleation events, leaving these questions unresolved (*20*–*22*). Current strategies rely on empirical tuning of global solution parameters—temperature (*23*), solvent (*24*), or additives (*25*, *26*)—that inevitably produce random polymorph distributions across substrates. Therefore, achieving polymorph control in deterministic spatial remains elusive (*27*, *28*).

Here, we present an innovative approach to spatially programmable polymorphic crystallization through microenvironment engineering. We designed a hierarchical silicon micropillar array platform that enables precise guidance of molecular self-assembly by systematically modulating local chemical parameters, including vapor distribution, interfacial chemistry, and spatial confinement effects. By creating distinct local chemical microenvironments, we achieved selective polymorphic control across different micropillar regions. To elucidate the intrinsic mechanisms of molecular self-assembly, we employed time-resolved grazing-incidence wide-angle x-ray scattering (GIWAXS) to track the real-time dynamics of molecular nucleation, Ostwald’s ripening, and polymorph formation. This method, for the first time, captured transient intermediate states and molecular-scale mechanisms of polymorphic transformations during stable crystal nucleus formation, providing critical scientific insights into complex molecular self-assembly processes. Based on this core methodology, we extended our regulation strategy to multiple molecular systems, successfully achieving programmable polymorphic formation across the entire visible spectrum (450–705 nm) with anisotropic polarized emission. On a single substrate, we integrated multidimensional polymorphic information including position, wavelength, intensity, and polarization, constructing a high-density optical information storage platform with an information density of 2^15^ bits/cm^2^, thereby opening new technological pathways for future optical information storage technologies.

## RESULTS

### Programming polymorph selection via microenvironmental regulation

**Fig. 1.**
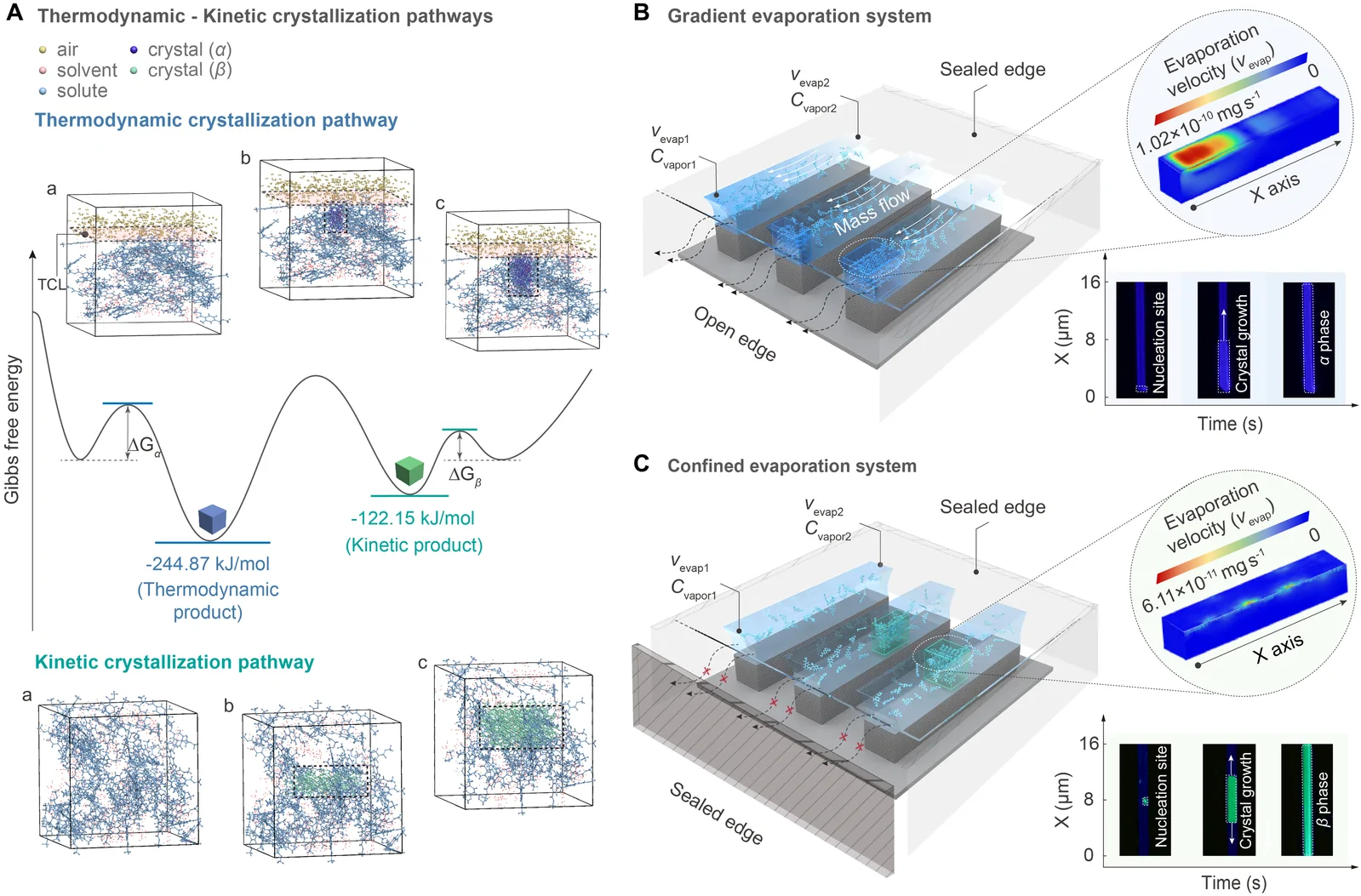
Programming polymorphic crystallization via microenvironmental regulation. (**A**) Free energy diagrams showing the nucleation mechanism of edge and bulk of microfluid via molecular dynamic (MD) simulations. (**B** and **C**) Schematic diagram depicting experimental setup of confined microfluid for tunable microenvironments: α-phase (blue) formation at preferential evaporating interface with asymmetric vapor distribution in gradient evaporation system. β-phase (cyan) formation at interior of microfluid with symmetric evaporation in confined evaporation system. (B and C) inset: In situ fluorescence microscopic observation of crystallization process of α-phase and β-phase. Zoom: CFD simulation of the solvent evaporation velocity at internal positions (along the *x*-axis) in microfluids as a function of the distance between interior and frontier.

Polymorphic crystallization is fundamentally governed by how molecules nucleate from solution—a process where solute molecules aggregate into dense, disordered clusters through localized concentration fluctuations, where multiple intermolecular forces cooperatively create pre-nucleation domains with enhanced molecular mobility. These clusters subsequently undergo structural reorganization, during which the competition between different intermolecular interactions guides the system toward specific polymorphic configurations (Fig. 1A, details in note S2). Using dimethylaminoterephthalate (DATP) molecules as a representative case, the free energy landscape revealed by molecular dynamics (MD) simulations illustrates two competing crystallization pathways: a thermodynamically driven route to the α-phase through a high nucleation barrier (leading to *G*_α_ = −244.87 kJ/mol), and a kinetically accessible route to the β phase via a lower barrier (reaching metastable *G*_β_ = −122.15 kJ/mol). Without external control over the local chemical environment, the system stochastically samples both pathways, rendering polymorph selection unpredictable (*29*, *30*). The outcome depends critically on local supersaturation and molecular transport kinetics. Under extreme supersaturation with directional solute flux, the system can overcome reorganization barriers to access the thermodynamically stable α phase despite its higher nucleation barrier. Conversely, moderate uniform supersaturation favors direct assembly into the kinetically accessible β phase, which becomes kinetically trapped.

These simulations suggest that engineering local chemical microenvironments—specifically controlling evaporation kinetics, and spatial confinement—could enable deterministic polymorph selection. Here, we designed two contrasting microfluidic configurations (Fig. 1, B and C and fig. S1). The gradient evaporation system exploits asymmetric vapor pressure to create extreme local supersaturation at the three-phase contact line (Fig. 1B and fig. S2). One end of the microchannel is exposed to ambient atmosphere while the opposite end remains closed, establishing vapor concentration asymmetry (*C*_vapor1_ > *C*_vapor2_). This drives directional solvent evaporation *v*_vapor1_ ≫ *v*_vapor2_, generating mass flow that continuously concentrates solute at the edge of microfluid. Time-resolved fluorescence microscopy captured the crystallization dynamics: intense blue emission (λ_em_ = 459 nm) emerged exclusively at the evaporation front, confirming site-specific α-phase nucleation (Fig. 1B, inset). The spatially localized pattern validates that gradient-driven extreme supersaturation activates the thermodynamic pathway.

The confined evaporation system maintains uniform vapor distribution to promote homogeneous nucleation (Fig. 1C and fig. S3). The closed-end geometry minimizes vapor escape, establishing symmetric conditions (*C*_vapor1_ ≈ *C*_vapor2_) that suppress asymmetric evaporation *v*_vapor1_ ≈ *v*_vapor2_. This evaporation-limited regime produces moderate supersaturation uniformly distributed across the solution volume. Time-resolved fluorescence imaging revealed cyan emission (λ_em_ = 505 nm) appearing homogeneously throughout the microchannel, indicating β-phase formation (Fig. 1C, inset). The spatially dispersed nucleation pattern confirms that suppression of concentration gradients kinetically traps the metastable polymorph.

### Construction of spatially differentiated microenvironments

**Fig. 2.**
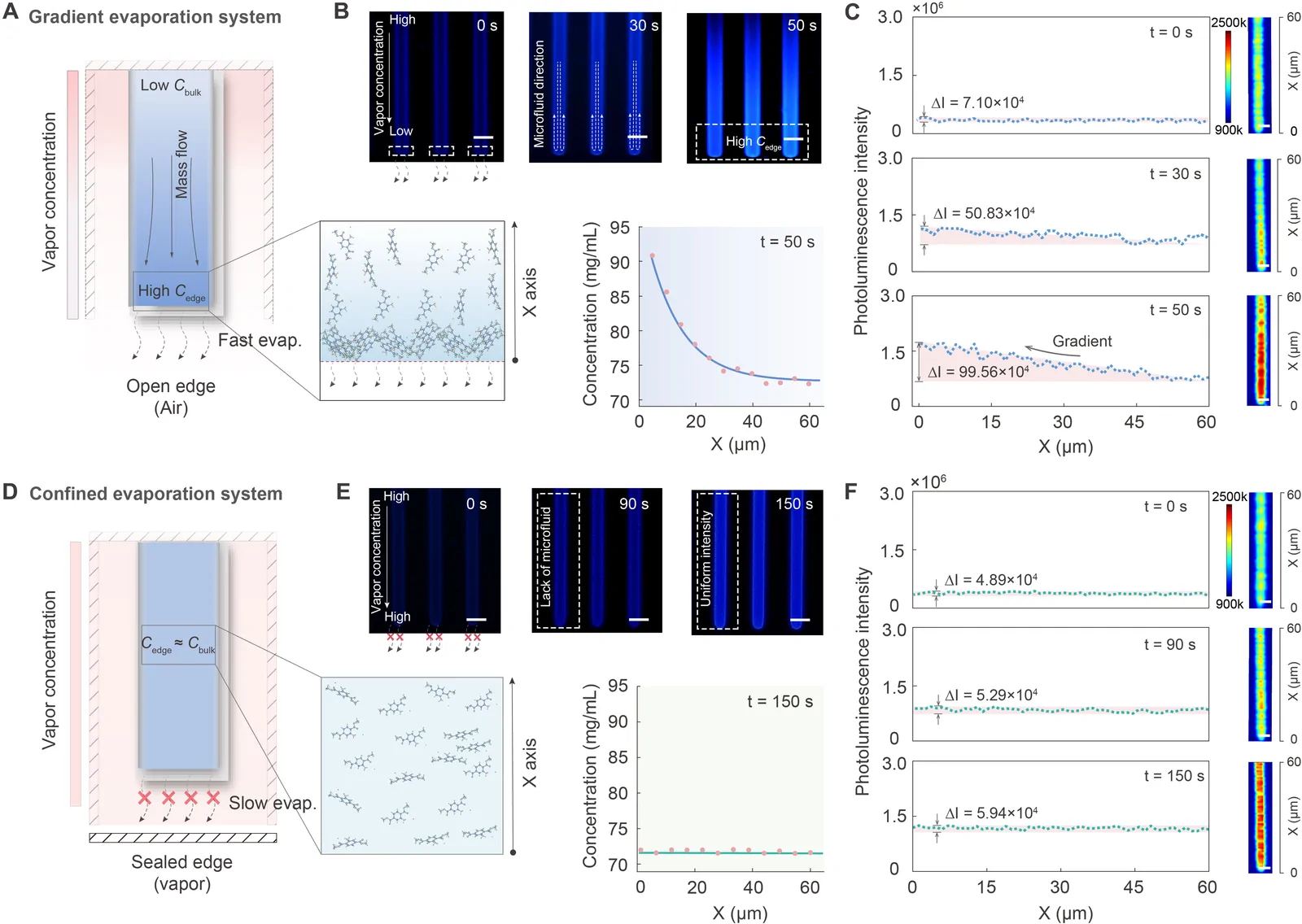
Construction of differentiated microenvironments. (**A** and **D**) Schematic illustration of (A) gradient evaporation system and (D) confined evaporation system. (**B** and **E**) In situ fluorescence microscopy images of the evolution of time-dependent evaporation dynamics, inset: The concentration at an internal position (along the X axis) in microfluid as a function of its distance from the frontier. Scale bars: 10 μm. (**C** and **F**) Time dependent fluorescence intensity evolution of the microfluid in (C) gradient evaporation system and (F) confined evaporation system as a function of its distance from the frontier (left) and its photoluminescence intensity mapping spectrum (right). Scale bars: 5 μm.

The divergent crystallization outcomes observed in gradient versus confined evaporation systems fundamentally arise from how solute molecules are spatially redistributed during evaporation—a process governed by the competition between capillary compensation flow and molecular diffusion (*31*–*34*). We combined computational fluid dynamics (CFD) simulations with time-resolved fluorescence microscopy to map concentration evolution from initial homogeneous distribution to the critical nucleation threshold (Fig. 2, note S3). For the gradient evaporation system, the asymmetric vapor distribution drives rapid evaporation at the edge of microfluid, with CFD simulations revealing evaporation flux *J*_evap_ = 1.02 × 10^−10^ mg·s^−1^ (Fig. 2A and fig. S4). This rapid evaporation generates capillary compensation flow that continuously transports solute-laden solution toward the evaporation front. Simultaneously, the resulting concentration gradient drives molecular diffusion in the opposite direction, attempting to re-equilibrate the concentration distribution. The balance between these competing transport mechanisms is quantified by the Péclet number *Pe* = (*v*·*L*)/*D*, where *v* represents flow velocity, *L* the characteristic length scale, and *D* the molecular diffusion coefficient (*35*, *36*)*.* Under gradient conditions, *Pe* > 1, indicating that advective transport overwhelms diffusive equilibration. This convection-dominated regime continuously enriches the evaporation front faster than back-diffusion can flatten the concentration profile, establishing steep spatial gradients with simulations predicting ∂*C*/∂*x* ∼ 0.9 mg·mL^−1^·mm^−1^ along the channel axis. Time-resolved fluorescence imaging experimentally validated this prediction (Fig. 2, B and C). At *t* = 0 s, emission intensity was spatially uniform (Δ*I* = 7.10 × 10^2^ a.u.), indicating homogeneous initial concentration. By *t* = 30 s, preferential enrichment at the edge of microfluid became evident (Δ*I* = 50.83 × 10^3^ a.u.). At *t* = 50 s, the concentration gradient intensified dramatically (Δ*I* = 99.56 × 10^5^ a.u.), with local concentration at the evaporation front reaching ∼40 mg/mL corresponding to supersaturation σ_local_ ∼ 3 (figs. S5 and S6). This extreme localized supersaturation, sustained by the advection-dominated transport regime, provides the thermodynamic driving force necessary to overcome the high α-phase nucleation barrier, explaining the site-specific blue emission crystal observed in Fig. 1B (fig. S7).

In contrast, the confined evaporation system suppresses evaporation by minimizing vapor escape. The closed-end geometry traps solvent vapor, establishing near-equilibrium vapor pressure that reduces evaporation flux to *J*_evap_ = 6.11 × 10^−11^ mg·s^−1^—more than 1.5-fold lower than gradient conditions (Fig. 2D). This suppressed evaporation drastically reduces capillary flow velocity, shifting the transport balance toward diffusion-dominated conditions with *Pe* ≪ 1. Under this regime, molecular diffusion effectively homogenizes any incipient concentration gradients on timescales much faster than evaporative concentration occurs. CFD simulations confirm ∂*C*/∂*x* < 0.01 mg·mL^−1^·mm^−1^, indicating that diffusion successfully maintains spatial uniformity. Supersaturation develops uniformly across the entire solution volume as slow evaporation gradually increases concentration everywhere simultaneously (fig. S4). Time-resolved fluorescence microscopy confirmed this spatially invariant evolution (Fig. 2, E and F, and fig. S8). Over 150 seconds, emission intensity increased uniformly throughout the microchannel, with concentration profiles remaining flat at ∼20 mg/mL everywhere—corresponding to moderate supersaturation σ ∼ 2. This uniform, moderate supersaturation activates only the low-barrier β-phase pathway, leading to cyan emission crystal throughout the channel (Fig. 1C, and fig. S9).

### Mechanistic origins of microenvironment-induced polymorph formation

**Fig. 3.**
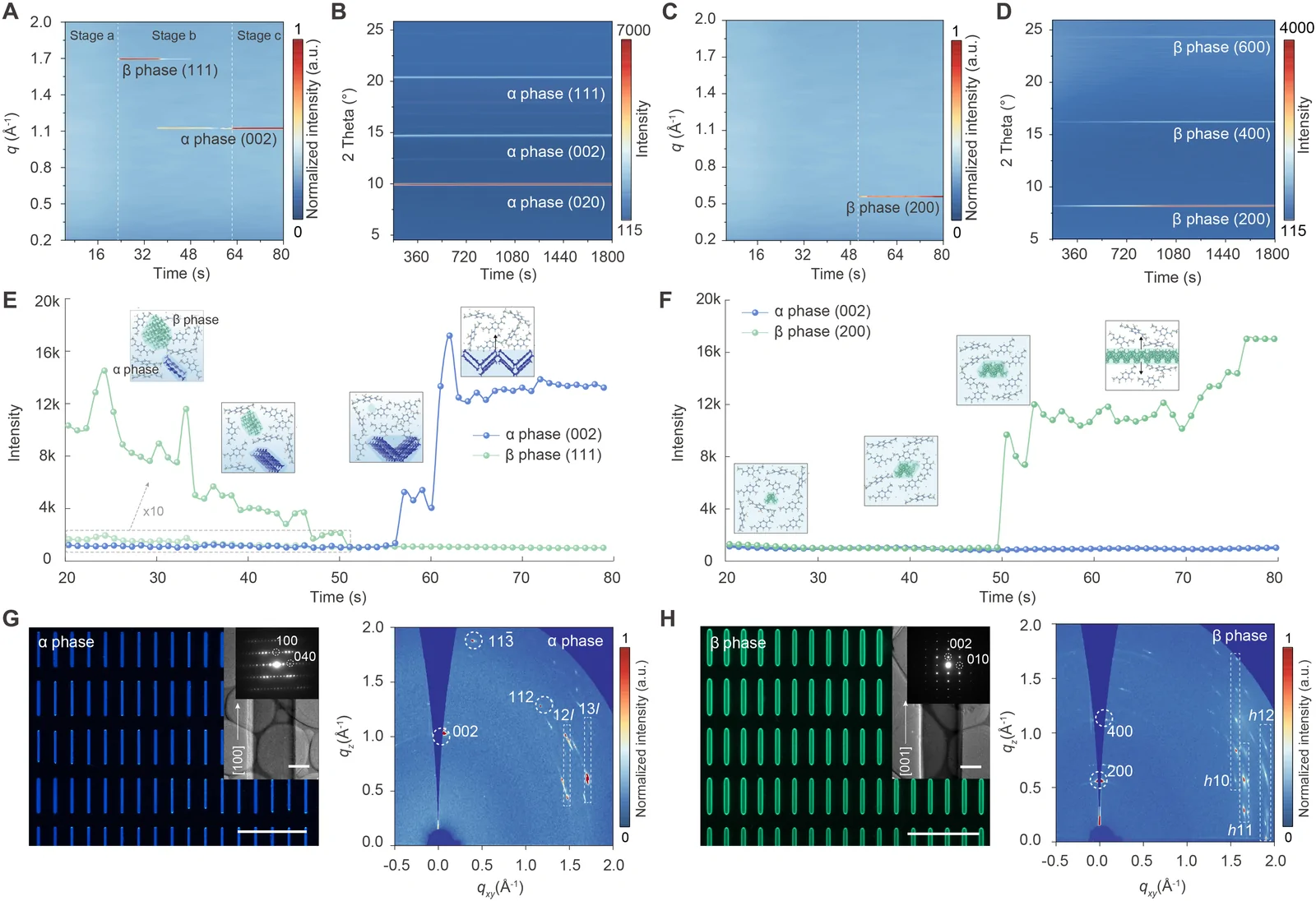
Microenvironment-induced polymorphic crystallization. (**A** and **C**) Radially integrated GIWAXS data as a function of *q* position for (A) α-phase and (C) β-phase during early stage of nucleation process. (**B** and **D**) Time-resolved XRD profiles for growth process of (B) α-phase and (D) β-phase. The color bar represents intensity. (**E** and **F**) Time-dependent intensity curve of characteristic diffraction peaks for GIWAXS of (E) α-phase and (F) β-phase. Inset: Schematic diagram depicting crystallization process through different pathways. (**G** and **H**) Fluorescence microscopy images of α-phase (G, left) and β-phase (H, left) arrays obtained, insets: selected area electron diffraction (SAED) patterns of polymorphism crystals, respectively. GIWAXS patterns of polymorphism arrays of DATP, further suggesting the ordered crystallographic orientation of microwire arrays, denoted as (G, right) α-phase and (H, right) β-phase, respectively. Scale bars, (G), (H), 100 μm, insets of (G), (H), 2 μm.

To directly observe the structural evolution from solution to crystalline polymorphs, we employed time-resolved GIWAXS to capture nucleation dynamics and time-resolved x-ray diffraction (XRD) to track subsequent growth (Fig. 3). These complementary techniques provide real-time molecular-scale structural snapshots under differentiated microenvironments.

For the gradient evaporation system, time-resolved GIWAXS revealed a sequential three-stage transformation during the nucleation phase (16–80 s, Fig. 3A and fig. S10). In stage a, diffuse scattering dominated with no crystalline peaks, indicating the absence of long-range-order crystalline structure at this early stage. At stage b (∼20–35 s), a distinct diffraction signal emerged at *q*_xy_ ∼ 1.7 Å^−1^ corresponding to β-phase (111) reflections, demonstrating initial nucleation via the metastable kinetic pathway. The intensity-time profile (Fig. 3E) shows β-phase (111) intensity rising to a maximum at ∼28 s before declining—a clear dissolution signature. Simultaneously, at stage c (∼30 s onward), a new reflection appeared at *q*_z_ ∼ 0.5 Å^−1^ corresponding to α-phase (002), which grew monotonically as β-phase signals diminished. By 50 s, α phase became the dominant species while β phase intensity approached baseline. This temporal sequence provides direct evidence for an Ostwald’s ripening, in which the α phase forms from an initially nucleated β phase rather than by direct nucleation under extreme supersaturation. Here, extreme local supersaturation promotes the initial formation of metastable β crystallites, while the sustained directional solute flux maintains the nonequilibrium chemical potential gradient that drives their dissolution and recrystallization into the thermodynamically stable α phase. During the subsequent growth phase, time-resolved XRD tracking revealed sustained α-phase crystallization (Fig. 3B). The time-resolved diffraction map shows persistent peaks at 2θ corresponding to α-phase (002), (020), and (111), with intensities increasing monotonically as crystal domains expanded. The continuous presence of only α-phase reflections throughout the observation confirms complete phase transformation with no β-phase remnants.

In stark contrast, the confined evaporation system exhibited direct nucleation without any phase transformation (Fig. 3, C and F, and fig. S11). Time-resolved 2D GIWAXS patterns captured during nucleation showed β-phase diffraction spots appearing and intensifying over time. Critically, no α-phase reflections emerged at any point during nucleation. During the growth phase, time-resolved XRD revealed sustained β-phase crystallization (Fig. 3D) with diffraction peaks at 2θ positions corresponding to (200), (400), and (600). These reflections grew monotonically throughout the observation window without any α-phase signatures appearing, demonstrating complete kinetic trapping in the metastable polymorph. The persistence of the metastable β phase under confined evaporation conditions is attributed to the moderate, spatially uniform supersaturation (σ ∼ 2; *Pe* ≪ 1) established by the diffusion-dominated transport regime, which inherently limits the density of nucleation events and yields fewer but larger crystals (*37*–*39*). Because the free energy of a crystal particle becomes size-dependent as surface and interfacial contributions grow at small dimensions, evaporation-driven crystallization in confined spaces can alter the apparent stability ranking of organic polymorphs (*40*–*42*). In our system, these larger crystallites possess an overall surface energy that is not as high as that of smaller crystals, providing insufficient thermodynamic driving force for dissolution and structural reorganization into the stable α phase. They therefore resist Ostwald transformation and become kinetically locked in the metastable β-phase. The concurrent absence of directional solute flux further suppresses the mass-transport pathways required for structural reorganization.

The final crystalline products demonstrate deterministic polymorph control with spatial organization (Fig. 3, G and H and figs. S12 and S13). Single-crystal arrays prepared under gradient conditions displayed ordered arrangements with intense blue fluorescence (λ_em_ = 459 nm, Fig. 3G, left). The inset shows transmission data and diffraction spots from individual single crystals within the array, confirming single-crystalline quality. Corresponding 2D GIWAXS exhibits sharp α-phase reflections, concentrated along specific azimuthal angles (Fig. 3G, right), confirming single-crystalline arrays with preferential crystallographic orientation. Similarly, arrays prepared under confined evaporation conditions exhibited cyan fluorescence (λ_em_ = 505 nm, Fig. 3H, left) with single-crystalline quality. 2D GIWAXS shows discrete β-phase diffraction spots with specific azimuthal angles (Fig. 3H, right), confirming preferential orientation.

These time-resolved structural measurements establish that microenvironmental control over supersaturation magnitude and transport kinetics determines polymorph transformations and formation. Gradient evaporation systems with extreme localized supersaturation promote initial nucleation through kinetically favored pathways, followed by phase transformation toward the thermodynamically stable state. Confined evaporation systems with moderate uniform supersaturation favor kinetic trapping in the initially nucleated metastable phase and suppress subsequent phase transformation. Such a contrast directly highlights the capacity of engineered microenvironments to selectively direct crystallization along competing free energy landscapes.

### Spatially programmed polymorphism through integrated microenvironments

**Fig. 4.**
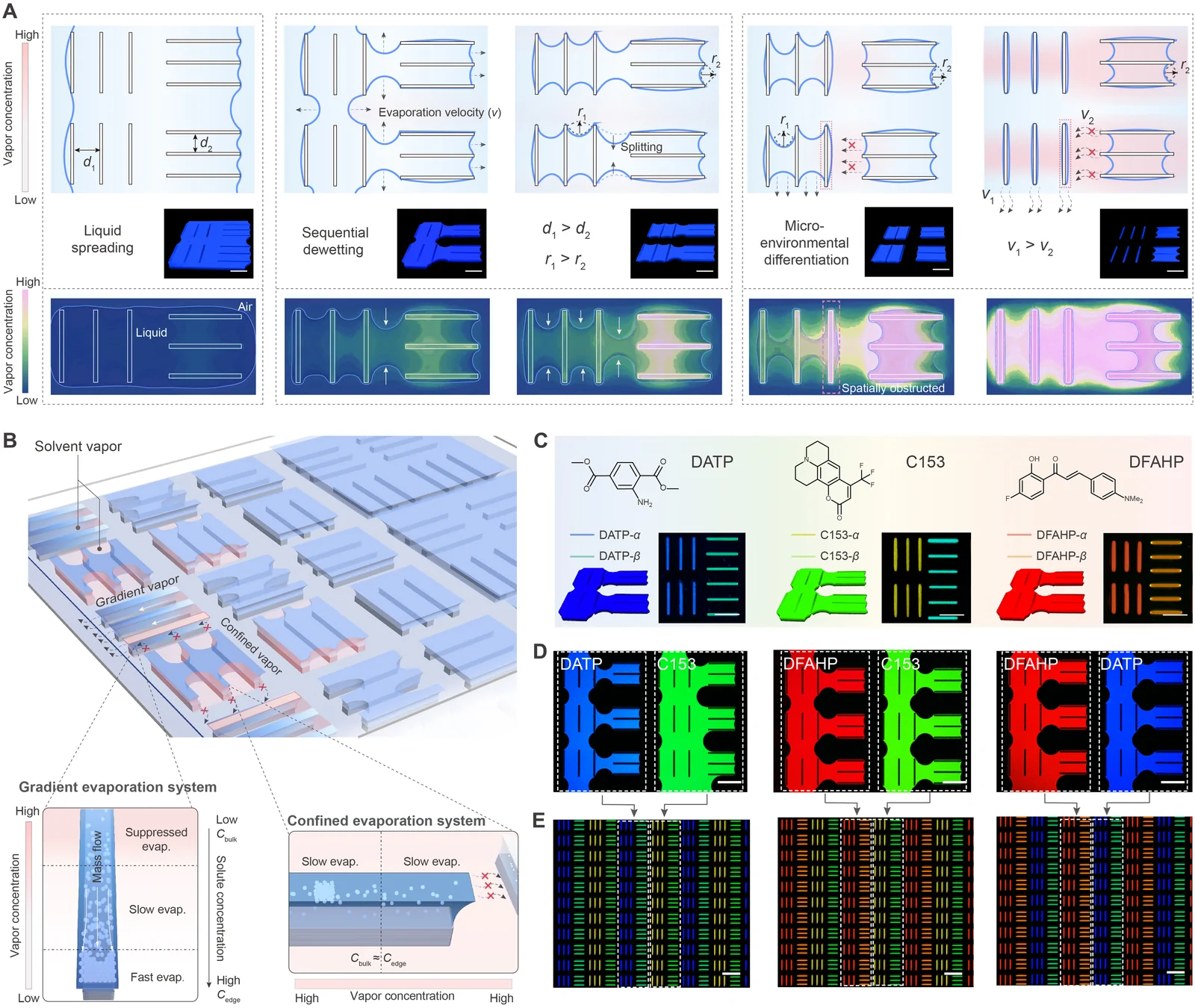
Spatial programmed polymorphic crystallization through microenvironmental integration. (**A**) Top panel: Schematic illustration of sequential dewetting process induced by the designed curvature radius difference (*d*_1_ = 24 μm, *d*_2_ = 20 μm). Middle panel: In situ confocal fluorescence microscopy observation of dewetting process. Bottom panel: CFD simulations revealing changes of solvent vapor concentration distribution during the dewetting process. The color bar represents solvent vapor-concentration fraction from low (blue) to high (red). (**B**) Schematic illustration of the integration of gradient evaporation/confined evaporation system with designed curvature radius difference. (i) Vertical micropillars (*d*_1_ = 24 μm) with bigger curvature radius constructing a gradient evaporation system, (ii) horizontal micropillars (*d*_2_ = 20 μm) with smaller curvature radius constructing a confined evaporation system by trapping vapor. (**C**) Confocal fluorescence microscopy (right) and fluorescence microscopy (left) of DATP, C153, DFAHP, respectively. (**D** and **E**) Binary-integrated molecular arrays: Confocal fluorescence microscopy (D) of and fluorescence microscopy (E) of DATP/C153, DFAHP/C153, DFAHP/DATP, respectively. Scale bars, (A), 30 μm, (C), 50 μm, (D), 50 μm, (E), 100 μm.

Having established that evaporation kinetics deterministically control polymorph selection, we designed a hierarchical silicon micropillar array platform that enables spatial programming of multiple coexisting microenvironments within a single substrate (Fig. 4, fig. S14). By systematically modulating channel curvature and spatial orientation (*d*_1_ > *d*_2_, *r*_1_ > *r*_2_), we engineered distinct local evaporation flux and vapor accumulation patterns that simultaneously generate predetermined polymorph arrays across different regions.

The key design principle exploits geometric control over vapor distribution (Fig. 4, A and B). Open-end vertical channels with large meniscus curvature maintain low local vapor pressure, allowing continuous vapor escape and high evaporation velocity. This creates steep supersaturation gradients (σ_local_ ∼ 3) that drive α-phase crystallization via Ostwald transformation. Conversely, closed-end horizontal channels with small curvature trap solvent vapor within confined volumes, establishing high local vapor pressure that suppresses evaporation. The accumulated vapor creates uniform, moderate supersaturation (σ_local_ ∼ 2) that nucleates and stabilizes metastable β phase. These gradient evaporation and confined evaporation systems coexist within the same device, separated by vapor barriers that prevent cross-contamination. Time-resolved confocal fluorescence microscopy and CFD simulations revealed five sequential stages during spatially differentiated crystallization (Fig. 4A). Initial uniform solution filling transitions to preferential dewetting in open channels while closed channels remain pinned (fig. S15). Vapor gathering establishes distinct concentration fields—high localized supersaturation in gradient regions (purple-pink regions) versus low uniform concentration in confined evaporation regions (cyan regions). Spatial differentiation of vapor diffusion stabilizes the two microenvironments, leading to selective crystallization: gradient regions produce α-phase single crystals with blue emission (459 nm) while confined evaporation regions generate β-phase arrays with cyan emission (505 nm).

To validate the generality of this approach, we selected two representative organic fluorophores with high photoluminescence quantum yields (>10%): coumarin-153 (C153) and 3-(4-(dimethylamino)phenyl)-1-(4-fluoro-2-hydroxyphenyl)prop-2-en-1-one (DFAHP) (fig. S16). Polymorphic single-crystal arrays of both compounds were assembled using identical templates. Low-magnification fluorescence microscopy confirmed the formation of large-area arrays exhibiting homogeneous crystal dimensions, well-defined morphologies, and precise spatial registration (Fig. 4C). Gradient evaporation channels selectively directed crystallization toward the C153 α-phase (green, 535 nm) and DFAHP α-phase (red, 705 nm), whereas confined evaporation channels yielded the C153 β-phase (yellow, 585 nm) and DFAHP β-phase (orange, 612 nm). Owing to the precise microenvironment control afforded by the template, fluorescence intensity uniformity across all polymorphic array units remained within 3%, and phase purity consistently exceeded 98% (details in fig. S17). Scanning electron microscopy (SEM) morphology and atomic force microscopy (AFM) topography further revealed flat crystal surfaces and well-defined boundaries, corroborating the high crystalline quality of the microstructure arrays (fig. S18). Extension of this strategy to additional organic fluorescent systems with chemically distinct molecular structures yielded equally precise, spatially programmed polymorphism in all cases (fig. S19).

The spatial independence of these microenvironments enables multicolor co-patterning by introducing different molecules into orthogonal regions (Fig. 4D and fig. S20). DATP-C153 combinations produced blue-green patterns, DFAHP-C153 yielded red-green arrays, and DFAHP-DATP generated red-blue architectures (Fig. 4E). The resulting patterns demonstrated excellent photostability over 100 consecutive readout cycles under 405 nm laser irradiation (22.3 mW cm^−2^), with no detectable emission peak shift, fluorescence intensity variation remaining within ±3%, and no measurable crystallinity degradation (fig. S21). These patterns maintain ∼100 μm spatial resolution with >500 individually addressable crystal units, demonstrating that vapor-distribution engineering provides scalable control over both polymorph selection and spatial organization without chemical modification or post-processing.

### Multidimensional optical encoding enabled by full-color polymorphic arrays

**Fig. 5.**
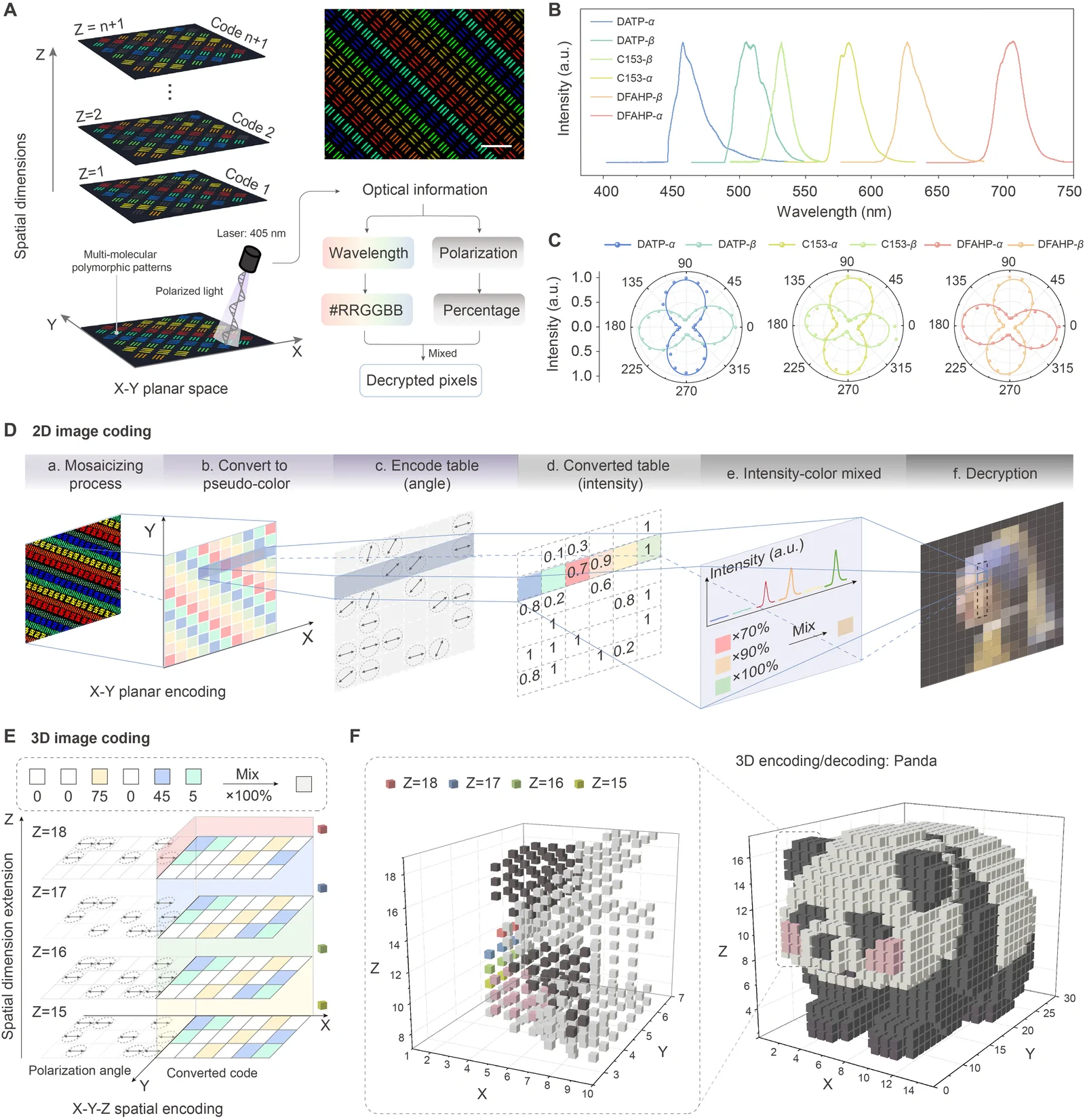
Multidimensional optical encryption based on full-color polymorphic arrays. (**A**) Schematic illustration of multi-dimensional encryption based on the extraction of optical information from hybrid polymorphic arrays of DATP/C153/DFAHP (upper right illustration). (B and C) UV-excited emission spectra (**B**) and polarized fluorescence profiles (**C**) of α/β phase of three organic molecules, respectively. (**D**) Schematic illustration of 2D encryption/decryption workflow in the X-Y plane, using an intensity-weighted hybrid algorithm to output the optical information of the six-color primitives as 2D pixel dots with color information. (**E**) Extended X-Y-Z 3D spatial encryption/decryption process. (**F**) 3D reconstruction of the Panda model (5,432 vertices), right zoomed inset showing the corresponding pixels of encoded positions (color points) in (E). Scale bar, (A), 200 μm.

Large-scale assembly of organic microcrystals into spatially defined patterns with multi-level information storage functionality is essential for next-generation high-density optical data storage (*43*, *44*). Our microenvironment engineering strategy enables precise fabrication of polymorphic molecular crystal arrays that achieve information encoding through two key mechanisms: (i) programmable 2D/3D spatial localization of information units and (ii) polarization-dependent fluorescence modulation (Fig. 5A). The template-guided arrays comprise vertically and horizontally aligned repeating cells, each acting as an information primitive, with individual polymorphic microarrays functioning as color pixels. The fluorescence emissions of the three molecules (DATP, C153, DFAHP) span the visible spectrum from 450 to 705 nm (Fig. 5B), enabling algorithmic Red-Green-Blue (RGB) blending to generate encoded images.

Polarization-resolved fluorescence measurements using a custom optical platform reveal distinct intensity-angle correlations for each polymorphic form across all six polymorphic-phase combinations (Fig. 5C), adding an additional storage dimension through polarization state. Each polymorph-polarization state functions as a distinct information primitive, where stored content is reconstructed by integrating the polarization profiles of all six polymorphic combinations. The system operates through a three-tiered transformation hierarchy (Fig. 5D): (i) angular modulation—polarization rotation from 0° to 90° produces fluorescence intensity gradients (*I*/*I*_0_ = 0–1); (ii) spectral encoding—RGB-weighted mixing algorithms combine six emission channels for color synthesis (fig. S22); (iii) spatial mapping—2D pixel arrays are generated via vector-field superposition. This multi-dimensional encoding strategy yields an information density of 2^15^ bits in proof-of-concept demonstrations, exemplified by a reconstructed microcrystal-based 2D image of Vermeer’s *Girl with a Pearl Earring*.

The storage platform extends to XYZ 3D space for volumetric image encoding and reconstruction (Fig. 5, E and F). In 3D information storage, each polarization-encoded layer displays a single slice of the 3D model, reconstructed by stacking slice information along the Z-direction (figs. S23 and S24). As a demonstration, a panda model is sliced into 18 layers for hierarchical information reconstruction (Fig. 5F). Image reconstruction is achieved by transcoding and retrieving each layer using RGB-weighted mixing algorithms, where eleven representative voxel sites on the panda’s face are highlighted with color-coded voxel cubes (Fig. 5E). The spatially layered stored information forms a five-dimensional storage platform comprising three spatial dimensions (X, Y, Z) combined with fluorescence polarization intensity and emission wavelength. Owing to the high reproducibility of large-area polymorphic crystal fabrication, this strategy should be extendable to higher-resolution image encoding and retrieval. Compared to fluorochromic polymer arrays and inkjet-printed dye systems, the polymorph-engineered platform also offers a unified architecture that combines spatial position, emission wavelength, and polarization state for multidimensional optical encoding, high-information-density storage, and anti-counterfeiting applications.

## DISCUSSION

In conclusion, this work developed a spatially programmable polymorphic crystallization system achieved through microenvironment design. This system is built upon two interconnected advances. First, through the use of time-resolved grazing-incidence x-ray scattering with sub-second resolution, the molecular-scale dynamics of polymorphic transformation during nucleation were directly captured. These observations reveal that a gradient microenvironment, characterized by extreme localized supersaturation and continuous solute flux, drives an Ostwald-step-rule-like progression from a kinetic polymorph to the thermodynamic product. In contrast, a uniform, confined microenvironment results in the direct nucleation and permanent kinetic trapping of a metastable phase. This mechanistic insight establishes a guiding framework that links local transport kinetics to polymorphic outcomes. Second, this understanding is translated into a general design principle via a silicon micropillar array platform engineered to create spatially distinct microenvironments—gradient evaporation zones and confined evaporation regions—on a single chip. This enables independent programming of the nucleation pathway in adjacent microscopic domains, achieving deterministic spatial patterning of multiple polymorphs from a common solution. The universality of this physical principle is demonstrated across three chemically distinct molecular systems, allowing for the spatial programming of six polymorphic forms whose emission colors span the entire visible spectrum (450–705 nm). Furthermore, by exploiting the polarization-dependent fluorescence of these oriented crystalline arrays, optical information is integrated across spatial, spectral, and polarization dimensions to create a high-density data storage platform with an information density of 2^15^ bits/cm^2^. This work establishes microenvironment engineering as a powerful and general paradigm, advancing the field from empirical bulk crystallization control toward the spatial programming of functional polymorphic matter.

## MATERIALS AND METHODS

### Materials

All chemicals were used without further purification. Trichloromethane (chloroform, ≥99.9%) were purchased from Sigma-Aldrich. Trimethoxy (1H,1H,2H,2H-heptadecafluorodecyl) silane (FAS, 98%) was purchased from Aladdin. Dimethylaminoterephthalate (DATP), coumarin-153 (C153) was purchased from Tokyo Chemical Industry Co. 3-(4-(Dimethylamino)phenyl)-1-(4-fluoro-2-hydroxyphenyl)prop-2-en-1-one (DFAHP) was synthesized according to previous reports (*45*).

### Fabrication of single-component polymorphism arrays

The organic molecules were dissolved in chloroform, to create solutions of varying concentration (20, 20 and 25 mg mL^−1^, for DATP, C153, DFAHP, respectively). Topographical templates were prepared by reactive ion etching followed by asymmetric-wettability modification (note S2 and fig. S1). In the gradient-evaporation system, 10 μL of the organic solution was carefully drop-cast onto an asymmetric-wettability topographical template. The glass substrate was then carefully placed on top of the topographical template, forming a “sandwich” structure. The assembly system was maintained at 25°C, and the solvent was allowed to evaporate for approximately 24 h. After complete solvent evaporation and removal of the topographical template, crystal arrays corresponding to the α phase were obtained. For the confined-evaporation system, 10 μL of the organic solution was carefully drop-cast onto the confined topographical template and covered with a glass substrate, forming a “sandwich” structure. The assembly system was also maintained at 25°C, and slow solvent evaporation proceeded for approximately 48 hours. After complete solvent evaporation and removal of the topographical template, crystal arrays corresponding to the β phase were obtained.

### Large-area photoluminescence spectra measurement

Large-area photoluminescence (PL) spectra were collected on a home-built optical microscopy. The PL signals were coupled to a grating spectrometer (Princeton Instrument, ARC-SP-2356), and images were recorded by a thermal-electrically cooled CCD (Princeton Instruments, PIX-256E). All optical measurements were performed at room temperature (25°C). Large-area photoluminescence measurements and polarization-dependent measurements were performed using a continuous-wave 405 nm laser with an excitation power of 22.3 mW cm^−2^ at the sample. Polarization-dependent fluorescence spectroscopy measurements were performed by rotating the polarizer in the optical path.

### Characterizations

The in situ fluorescent micrographs were collected on an optical microscope (Vision Engineering Co., UK) integrated with a charge-coupled device (CCD) camera. Fluorescence 3D reconstruction images were obtained by Confocal Microscope (Nikon, A1). UV-visible absorption spectra were obtained by using a Cary 5000 UV-vis-NIR spectrophotometer. Fluorescence spectra were recorded using an F-4500 fluorescence spectrophotometer (Hitachi, Japan) with an excitation wavelength of 405 nm and an excitation power of 22.3 mW cm^−2^. The PLQY and TRPL decay curves of the powder samples were recorded using the Edinburgh instrument FLS 1000 with an excitation wavelength of 405 nm. AFM photographs were collected by a Bruker MultiMode 8 Atomic Force Microscope. Contact angles were measured on a Dataphysics OCA20 contact-angle system. SEM images were acquired by SEM (Hitachi, S-4800, Japan) at an accelerating voltage of 5.0 kV and a beam current of 10 μA. XRD patterns was collected by x-ray diffractometer (Bruker, D8 focus, Germany) with monochromatized Cu Kα radiation (λ = 1.5406 Å). GIWAXS patterns were collected at the Shanghai Synchrotron Radiation Facility of BL15U1 (SSRF-BL15U1). TEM (JEOL, 2100, Japan) and SAED were obtained with an accelerating voltage of 200 kV.

## Supplementary Materials

### This PDF file includes:

Notes S1 to S3 Figs. S1 to S25

## References

[1] O. Sachnik, X. Tan, D. Dou, C. Haese, N. Kinaret, K.-H. Lin, D. Andrienko, M. Baumgarten, R. Graf, G.-J. A. H. Wetzelaer, J. J. Michels, P. W. M. Blom, Elimination of charge-carrier trapping by molecular design. Nat. Mater. 22, 1114–1120 (2023). 37386064 10.1038/s41563-023-01592-3 PMC10465354

[2] Z. Qin, C. Gao, H. Gao, T. Wang, H. Dong, W. Hu, Molecular doped, color-tunable, high-mobility, emissive, organic semiconductors for light-emitting transistors. Sci. Adv. 8, eabp8775 (2022). 35857474 10.1126/sciadv.abp8775 PMC9269892

[3] T. Okamoto, S. Kumagai, E. Fukuzaki, H. Ishii, G. Watanabe, N. Niitsu, T. Annaka, M. Yamagishi, Y. Tani, H. Sugiura, T. Watanabe, S. Watanabe, J. Takeya, Robust, high-performance n-type organic semiconductors. Sci. Adv. 6, eaaz0632 (2020). 32494668 10.1126/sciadv.aaz0632 PMC7195148

[4] T. Furukawa, N. Kasuya, H. Takayanagi, S. Watanabe, J. Takeya, Hall mobility exceeding 100 cm^2^ v^-1^ s^-1^ observed in strained organic semiconductors. Sci. Adv. 11, eaea1634 (2025). 41032610 10.1126/sciadv.aea1634 PMC12487896

[5] Z. Xie, D. Liu, C. Gao, H. Dong, W. Hu, High-mobility emissive organic semiconductors: An emerging class of multifunctional materials. Nat. Rev. Mater. 9, 837–839 (2024).

[6] A. Yi, S. Chae, H. M. Luong, S. H. Lee, H. Lee, H. Yoon, D.-H. Kim, H. J. Kim, T.-Q. Nguyen, Room-temperature-processed perovskite solar cells surpassing 24% efficiency. Joule 8, 2087–2104 (2024).

[7] L. Zhang, X. Zhong, E. Pavlica, S. Li, A. Klekachev, G. Bratina, T. W. Ebbesen, E. Orgiu, P. Samorì, A nanomesh scaffold for supramolecular nanowire optoelectronic devices. Nat. Nanotechnol. 11, 900–906 (2016). 27454879 10.1038/nnano.2016.125

[8] A. S. Tayi, A. Kaeser, M. Matsumoto, T. Aida, S. I. Stupp, Supramolecular ferroelectrics. Nat. Chem. 7, 281–294 (2015). 25803466 10.1038/nchem.2206

[9] P. Shi, Y. Ding, B. Ding, Q. Xing, T. Kodalle, C. M. Sutter-Fella, I. Yavuz, C. Yao, W. Fan, J. Xu, Y. Tian, D. Gu, K. Zhao, S. Tan, X. Zhang, L. Yao, P. J. Dyson, J. L. Slack, D. Yang, J. Xue, M. K. Nazeeruddin, Y. Yang, R. Wang, Oriented nucleation in formamidinium perovskite for photovoltaics. Nature 620, 323–327 (2023). 37344595 10.1038/s41586-023-06208-z

[10] R. Ji, Z. Zhang, Y. J. Hofstetter, R. Buschbeck, C. Hänisch, F. Paulus, Y. Vaynzof, Perovskite phase heterojunction solar cells. Nat. Energy 7, 1170–1179 (2022).

[11] G. R. Desiraju, Cryptic crystallography. Nat. Mater. 1, 77–79 (2002). 12618812 10.1038/nmat726

[12] A. E. S. Van Driessche, N. Van Gerven, P. H. H. Bomans, R. R. M. Joosten, H. Friedrich, D. Gil-Carton, N. Sommerdijk, M. Sleutel, Molecular nucleation mechanisms and control strategies for crystal polymorph selection. Nature 556, 89–94 (2018). 29620730 10.1038/nature25971

[13] Q. Gao, J. Ai, S. Tang, M. Li, Y. Chen, J. Huang, H. Tong, L. Xu, L. Xu, H. Tanaka, P. Tan, Fast crystal growth at ultra-low temperatures. Nat. Mater. 20, 1431–1439 (2021). 33958770 10.1038/s41563-021-00993-6

[14] S. Pratap, F. Babbe, N. S. Barchi, Z. Yuan, T. Luong, Z. Haber, T.-B. Song, J. L. Slack, C. V. Stan, N. Tamura, C. M. Sutter-Fella, P. Müller-Buschbaum, Out-of-equilibrium processes in crystallization of organic-inorganic perovskites during spin coating. Nat. Commun. 12, 5624 (2021). 34561460 10.1038/s41467-021-25898-5 PMC8463609

[15] G. Giri, R. Li, D. M. Smilgies, E. Q. Li, Y. Diao, K. M. Lenn, M. Chiu, D. W. Lin, R. Allen, J. Reinspach, S. C. Mannsfeld, S. T. Thoroddsen, P. Clancy, Z. Bao, A. Amassian, One-dimensional self-confinement promotes polymorph selection in large-area organic semiconductor thin films. Nat. Commun. 5, 3573 (2014). 24736391 10.1038/ncomms4573

[16] Y. Liu, Z. Wang, Y. Zhang, T. Wu, T. Zheng, B. Guo, G. Reiter, J. Xu, The size of critical secondary nuclei of polymer crystals does not depend on supersaturation. Nat. Commun. 16, 3773 (2025). 40263380 10.1038/s41467-025-58962-5 PMC12015502

[17] J. Zhang, Y. Yang, W. Li, Z. Tang, Z. Hu, H. Wei, J. Zhang, B. Yang, Precise arraying of perovskite single crystals through droplet-assisted self-alignment. Sci. Adv. 10, eado0873 (2024). 38985869 10.1126/sciadv.ado0873 PMC11235166

[18] M. Fitzner, G. C. Sosso, F. Pietrucci, S. Pipolo, A. Michaelides, Pre-critical fluctuations and what they disclose about heterogeneous crystal nucleation. Nat. Commun. 8, 2257 (2017). 29273707 10.1038/s41467-017-02300-x PMC5741629

[19] D. Firaha, Y. M. Liu, J. van de Streek, K. Sasikumar, H. Dietrich, J. Helfferich, L. Aerts, D. E. Braun, A. Broo, A. G. DiPasquale, A. Y. Lee, S. Le Meur, S. O. Nilsson Lill, W. J. Lunsmann, A. Mattei, P. Muglia, O. D. Putra, M. Raoui, S. M. Reutzel-Edens, S. Rome, A. Y. Sheikh, A. Tkatchenko, G. R. Woollam, M. A. Neumann, Predicting crystal form stability under real-world conditions. Nature 623, 324–328 (2023). 37938708 10.1038/s41586-023-06587-3 PMC10632141

[20] J. S. Du, Y. Bae, J. J. De Yoreo, Non-classical crystallization in soft and organic materials. Nat. Rev. Mater. 9, 229–248 (2024).

[21] J. Zhou, Y. Yang, Y. Yang, D. S. Kim, A. Yuan, X. Tian, C. Ophus, F. Sun, A. K. Schmid, M. Nathanson, H. Heinz, Q. An, H. Zeng, P. Ercius, J. Miao, Observing crystal nucleation in four dimensions using atomic electron tomography. Nature 570, 500–503 (2019). 31243385 10.1038/s41586-019-1317-x

[22] G. Zhan, Z. F. Cai, K. Strutynski, L. Yu, N. Herrmann, M. Martinez-Abadia, M. Melle-Franco, A. Mateo-Alonso, S. Feyter, Observing polymerization in 2D dynamic covalent polymers. Nature 603, 835–840 (2022). 35355001 10.1038/s41586-022-04409-6

[23] L. Wu, S. Hu, F. Yang, G. Li, J. Wang, W. Zuo, J. J. Jerónimo-Rendon, S.-H. Turren-Cruz, M. Saba, M. Saliba, M. K. Nazeeruddin, J. Pascual, M. Li, A. Abate, Resilience pathways for halide perovskite photovoltaics under temperature cycling. Nat. Rev. Mater. 10, 536–549 (2025).

[24] X. Li, J. Wang, T. Wang, N. Wang, S. Zong, X. Huang, H. Hao, Molecular mechanism of crystal nucleation from solution. Sci. China Chem. 64, 1460–1481 (2021).

[25] Y. Iida, S. Hiraide, S. Watanabe, Additive strategy for nucleation pathway control based on the understanding of molecular size effect on crystallization. J. Chem. Phys. 162, 214503 (2025). 40454592 10.1063/5.0266286

[26] J. Nozawa, M. Sato, S. Uda, K. Fujiwara, Polymorphic transitions during nonclassical nucleation and growth in the colloidal heteroepitaxy. Commun. Phys. 8, 134 (2025).

[27] A. Perevedentsev, M. Campoy-Quiles, Rapid and high-resolution patterning of microstructure and composition in organic semiconductors using ‘molecular gates’. Nat. Commun. 11, 3610 (2020). 32680991 10.1038/s41467-020-17361-8 PMC7367850

[28] M. Li, Y. Chen, H. Tanaka, P. Tan, Revealing roles of competing local structural orderings in crystallization of polymorphic systems. Sci. Adv. 6, eaaw8938 (2020). 32656336 10.1126/sciadv.aaw8938 PMC7329355

[29] A. J. Cruz-Cabeza, J. Bernstein, Conformational polymorphism. Chem. Rev. 114, 2170–2191 (2014). 24350653 10.1021/cr400249d

[30] E. Pretti, H. Zerze, M. Song, Y. Ding, R. Mao, J. Mittal, Size-dependent thermodynamic structural selection in colloidal crystallization. Sci. Adv. 5, eaaw5912 (2019). 31548983 10.1126/sciadv.aaw5912 PMC6744263

[31] W. Deng, X. Zhang, H. Dong, J. Jie, X. Xu, J. Liu, L. He, L. Xu, W. Hu, X. Zhang, Channel-restricted meniscus self-assembly for uniformly aligned growth of single-crystal arrays of organic semiconductors. Mater. Today 24, 17–25 (2019).

[32] J. Peng, C. Q. Xia, Y. Xu, R. Li, L. Cui, J. K. Clegg, L. M. Herz, M. B. Johnston, Q. Lin, Crystallization of CsPbBr_3_ single crystals in water for X-ray detection. Nat. Commun. 12, 1531 (2021). 33750768 10.1038/s41467-021-21805-0 PMC7943776

[33] J. Zhong, D. Zhou, Q. Bai, C. Liu, X. Fan, H. Zhang, C. Li, R. Jiang, P. Zhao, J. Yuan, X. Li, G. Zhan, H. Yang, J. Liu, X. Song, J. Zhang, X. Huang, C. Zhu, C. Zhu, L. Wang, Growth of millimeter-sized 2D metal iodide crystals induced by ion-specific preference at water-air interfaces. Nat. Commun. 15, 3185 (2024). 38609368 10.1038/s41467-024-47241-4 PMC11014996

[34] H. Han, S. Kallakuri, Y. Yao, C. B. Williamson, D. R. Nevers, B. H. Savitzky, R. S. Skye, M. Xu, O. Voznyy, J. Dshemuchadse, L. F. Kourkoutis, S. J. Weinstein, T. Hanrath, R. D. Robinson, Multiscale hierarchical structures from a nanocluster mesophase. Nat. Mater. 21, 518–525 (2022). 35422509 10.1038/s41563-022-01223-3

[35] F. Boulogne, F. Ingremeau, H. A. Stone, Coffee-stain growth dynamics on dry and wet surfaces. J. Phys. Condens. Matter 29, 074001 (2017). 28035085 10.1088/1361-648X/aa5160

[36] J. K. Sun, Y. I. Sobolev, W. Zhang, Q. Zhuang, B. A. Grzybowski, Enhancing crystal growth using polyelectrolyte solutions and shear flow. Nature 579, 73–79 (2020). 32132690 10.1038/s41586-020-2042-1

[37] J. L. Hilden, C. E. Reyes, M. J. Kelm, J. S. Tan, J. G. Stowell, K. R. Morris, Capillary precipitation of a highly polymorphic organic compound. Cryst. Growth Des. 3, 921–926 (2003).

[38] B. D. Hamilton, M. A. Hillmyer, M. D. Ward, Glycine polymorphism in nanoscale crystallization chambers. Cryst. Growth Des. 8, 3368–3375 (2008).

[39] B. D. Hamilton, J.-M. Ha, M. A. Hillmyer, M. D. Ward, Manipulating crystal growth and polymorphism by confinement in nanoscale crystallization chambers. Acc. Chem. Res. 45, 414–423 (2012). 22035061 10.1021/ar200147v

[40] N. Fellah, I. J. C. Dela Cruz, B. G. Alamani, A. G. Shtukenberg, A. V. Pandit, M. D. Ward, A. S. Myerson, Crystallization and polymorphism under nanoconfinement. Cryst. Growth Des. 24, 3527–3558 (2024).

[41] G. T. Rengarajan, D. Enke, M. Steinhart, M. Beiner, Size-dependent growth of polymorphs in nanopores and Ostwald’s step rule of stages. Phys. Chem. Chem. Phys. 13, 21367–21374 (2011). 22033648 10.1039/c1cp22679g

[42] K. Zhang, N. Fellah, V. López-Mejías, M. D. Ward, Polymorphic phase transformation pathways under nanoconfinement: Flufenamic acid. Cryst. Growth Des. 20, 7098–7103 (2020).

[43] S. Nocentini, U. Rührmair, M. Barni, D. S. Wiersma, F. Riboli, All-optical multilevel physical unclonable functions. Nat. Mater. 23, 369–376 (2024). 38191630 10.1038/s41563-023-01734-7

[44] X. Ouyang, Y. Xu, M. Xian, Z. Feng, L. Zhu, Y. Cao, S. Lan, B.-O. Guan, C.-W. Qiu, M. Gu, X. Li, Synthetic helical dichroism for six-dimensional optical orbital angular momentum multiplexing. Nat. Photonics 15, 901–907 (2021).

[45] X. Cheng, Y. Zhang, S. Han, F. Li, H. Zhang, Y. Wang, Multicolor amplified spontaneous emissions based on organic polymorphs that undergo excited-state intramolecular proton transfer. Chem. A Eur. J. 22, 4899–4903 (2016). 10.1002/chem.201600355 26917274

