# Supplementary material for: Spatially programmed polymorphic crystallization via microenvironment design
Source: Sci Adv. 2026 Sep 11;12(37):eaeg8728. doi: 10.1126/sciadv.aeg8728 (PMC13564892; doi:10.1126/sciadv.aeg8728)
Supplement: Supplementary file 1 [file sciadv.aeg8728_sm.pdf]

Science Advances
AAAS

Supplementary Materials for

Spatially programmed polymorphic crystallization via microenvironment design

Zhenglian Qin *et al.*

Corresponding author: Xiao Wei, weixiao16@mails.jlu.edu.cn; Wen Wen, wen24@ustc.edu.cn; Yuchen Wu, wuyuchen@iccas.ac.cn

Sci. Adv. **12**, eaeg8728 (2026)
DOI: 10.1126/sciadv.aeg8728

This PDF file includes:

Notes S1 to S3
Figs. S1 to S25

Note S1. Selective modification to fabricate asymmetrically-wettable topographical templates.

Asymmetric-wettability modification of topographical templates. The pristine topographical template with symmetric wettability was cleaned with deionized water, ethanol and isopropanol followed by dealing with oxygen plasma for removing the contaminants on surface. We carried out selective modification of topographical template by covering a SU-8 protection layer onto the tops of micropillars. The SU-8 layer was slightly covered onto a flat silicon substrate and then pressed onto tops of micropillars under the heating of 70 °C. A thin layer of SU-8 was coated onto tops of micropillars after peeling off the silicon substrate. A low surface-energy molecule, heptadecafluorodecyltrimethoxysilane (FAS), was harnessed to selectively modify the sidewalls and bottoms of micropillars. For FAS modification, the top-protected topographical templates were placed into a glass culture dish followed by adding 20 μL of FAS liquid. The glass culture dish was silanized by FAS molecules in a decompression environment at room temperature for 24 h and then heated at 90 °C for 2h, resulting in a homogeneous highly lyophobic surface at bottom and sidewall regions of micropillars. After FAS modification, the SU8 layer was removed by acetone, yielding asymmetric-wettability topographical templates. The sidewall regions show a high CA of 94.0 ± 0.5° with lyophobic nature, while the tops maintain the lyophilic nature with a contact angle of 13.0 ± 0.5° (details in fig. S1).

Note S2. Molecular dynamics simulations.

The molecular orbitals of the molecules were calculated by density functional theory (B3LYP-D3BJ//6-31G(d, p)). The growth morphologies of single crystals were simulated using Materials Studio software, based on attachment energy theory. Optimization of nitrogen molecules, DCM molecules and solute molecules using the Geometry Optimization module of the Dmol3 module, method: PBE potential function using the Generalized Gradient Approximation Generalized Gradient Approximation, with a truncation energy of $1.0e^{-5}$ Ha, truncation stress 0.002 Ha/A, truncation displacement 0.005 A, and a maximum cycle length of 500. The truncation displacement is 0.005 A, the energy convergence criterion is $1.0e^{-6}$ Ha, and the maximum number of cycles is 500. Optimize the given two crystal models A and B using Castep's Geometry Optimization module by using the PBE potential function with a Generalized Gradient Approximation (GGA) with an energy cutoff of 571.40 eV, a cutoff stress of 0.03 eV/A, and a critical pressure of 0.05 GPa. The energy cutoff is 571.40 eV, the cutoff stress is 0.03 eV/A, the cutoff pressure is 0.05 GPa, the cutoff displacement is 0.001 A, the energy convergence criterion is $1.0e^{-6}$ eV, the maximum

number of cycles is 200, the convergence algorithm is the LBGFS function, and the pseudo-potential is the OTFG ultrasoft. Use the Amorphous cell module to build the initial low concentration and high concentration solution model, in which the crystals produced in the high concentration system are A, and the crystals produced in the low concentration system are B; the crystallization process of high concentration is studied by building the interface between nitrogen and solution system, and the crystallization process of low concentration is studied by building the pure solution system. Use the Dynamic module of Forcite to simulate the molecular dynamics of the initial state of the two models and the different states of evaporation and crystallization, a total of eight groups, the synthesis system is the NVT synthesis system, the step size is 1 fs, the total equilibrium time is 500 ps, the total number of simulation steps is 500,000, and the output of a snapshot of the trajectory is outputted in every 5,000 steps, the temperature control function is Nose, and the Q value is 0.01 The cut-off radius of the van der Waals force is 18.5 A, the force field is COMPASSIII, and the temperature is 298.15 K. The simulation allows the system to reach an energetically stable state and extracts the end-states a1-a8 and calculates the potential energies E_1-E_8, which are a1-a4 for the high-concentration system, and a5-a8 for the low-concentration system. In particular, in order to maintain the conservation of internal energy of the matter, the a2-a4 and a6 -a8 models need to add the potential energy of DCM reduced by evaporation when calculating the potential energy, so as to obtain eight potential energy values of E_1-E_8. The high concentration system 1-4 crystallization free energy $E_{x'} = E_x - E_1 - E_{solvation}$; the low concentration system 5-8 crystallization free energy $E_{x'} = E_x - E_5 - E_{solvation}$, obtaining eight free energy $E_{1'}$-$E_{8'}$, where the initial state $E_{1'}=E_{5'}=0$.

Note S3. Theoretical derivation of the spatial and temporal variations of vapor concentration in de-wetting process

In a gradient evaporation system, the solvent evaporation process at the gas-liquid interface of the microfluidic front can be regarded as one-dimensional semi-infinite non-steady diffusion in free space. A simple geometric model is established: the evaporating liquid surface is located at x = 0, and the vapor freely diffuses to x tends to positive infinity, so the diffusion of vapor can be simplified as:

$$\frac{\partial C}{\partial t} = D\frac{\partial^2 C}{\partial x^2}$$

The boundary conditions are: $C(0, t) = C_s$; $C(\infty, t)=0$:

$$C(x, t) = C_s \cdot \mathrm{erfc}\left(\frac{x}{2\sqrt{Dt}}\right)$$

where erfc(z) is the complementary error function. Thus, the concentration gradient at

the liquid surface is:

$$\left.\frac{\partial C}{\partial x}\right|_{x=0} = C_{\mathrm{sat}} \cdot \frac{\mathrm{d}}{\mathrm{d}x}\operatorname{erfc}\left(\frac{x}{2\sqrt{Dt}}\right)\Bigg|_{x=0}$$

Derivation of the complementary error function gives:

$$\left.\frac{\partial C}{\partial x}\right|_{x=0} = -\frac{C_{\mathrm{sat}}}{\sqrt{\pi D t_0}}$$

According to Fick's first law, the rate of solvent volatilization at the gas-liquid interface of vertical groups is:

$$J_{\mathrm{ver}} = -D\left.\frac{\partial C}{\partial x}\right|_{x=0} = D\frac{C_{\mathrm{sat}}}{\sqrt{\pi D t_0}}$$

We establish the following two models to calculate the volatilization of the vertical and horizontal groups separately.

The first one is that in the vertical group, there is no obstruction in front of the evaporating liquid surface and the volatilization is free in free space, which can be considered as one-dimensional unsteady diffusion.

In the other model, the evaporating liquid surface front in the horizontal group is obstructed by the vertical group and the vapor accumulates on the walls of the obstruction, thus requiring a two-dimensional diffusion correction. In steady state, the diffusion equation degenerates into the Laplace equation:

$$\frac{\partial^2 C}{\partial x^2} + \frac{\partial^2 C}{\partial y^2} = 0$$

Expanded using Fourier series:

$$\mathrm{C(x, y)} = \sum_{\mathrm{n}=1}^{\infty} \frac{4C_{\mathrm{sat}}}{\mathrm{n}\pi} \sin\left(\frac{\mathrm{n}\pi D_3}{4H}\right) \frac{\cosh\left(\frac{\mathrm{n}\pi(L-x)}{2H}\right)}{\cosh\left(\frac{\mathrm{n}\pi L}{2H}\right)} \cos\left(\frac{\mathrm{n}\pi y}{2H}\right)$$

Here D_3 is the width of the interval between the vertical and horizontal groups, H is the distance between the substrate and the bottom of the silicon micropillar gap, and L is the distance from the evaporating liquid surface along the x direction.

At liquid surface (x = 0, y = 0), the diffusive flux J_{hor} is (dominant mode

approximation, n = 1):

$$J_{\mathrm{hor}} \approx \frac{2DC_{\mathrm{sat}}}{H} \sin\left(\frac{\pi D_3}{4H}\right) \tanh\left(\frac{\pi L}{2H}\right)$$

In our system, D_3 = 30 μm, H ≈ 15.5 μm:

$$J_{\mathrm{hor}} \approx \frac{4DC_{\mathrm{sat}}}{31} \cdot \tanh\left(\frac{\pi L}{31}\right)$$

Namely:

$$J_{\mathrm{hor}} = \frac{DC_{\mathrm{sat}}}{L_{\mathrm{e}}}$$

Where L_e represent equivalent diffusion lengths, $L_{\mathrm{e}} = \frac{31}{4\tanh\left(\frac{\pi L}{31}\right)}$.

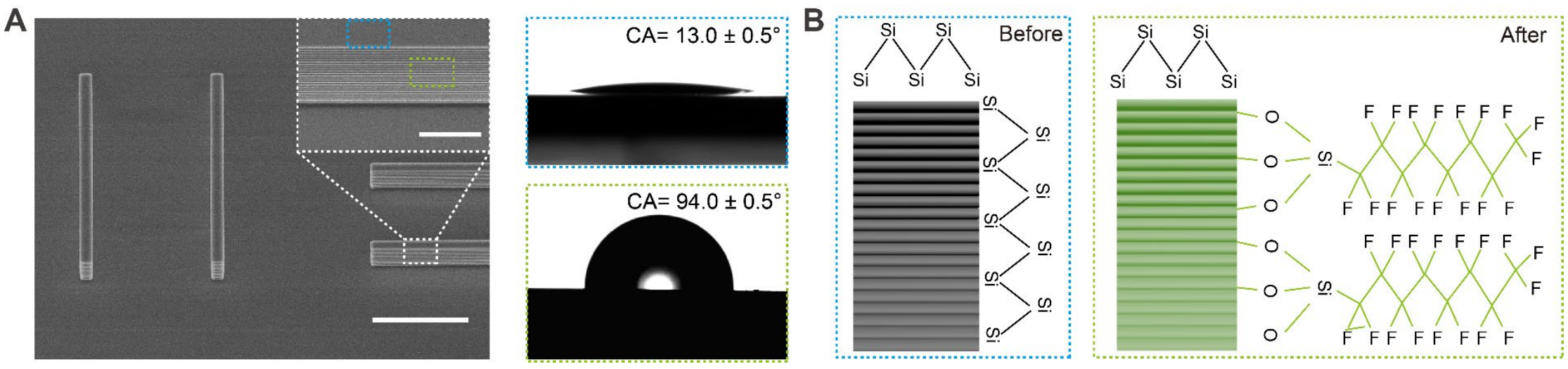

Fig. S1.
Selective modification to fabricate asymmetrically-wettable topographical templates. (**A**) The successful modification was validated by contact angle (CA) measurements. The sidewall regions show a high CA of 94.0 ± 0.5° with lyophobic nature, while the tops maintain the lyophilic nature with a contact angle of 13.0 ± 0.5°. (**B**) Schematic illustration comparing the hydrophilic and hydrophobic properties of the template surface before and after selective modification. The tops of the micropillars are hydrophilic, while the sidewalls are modified with FAS to be hydrophobic. Scale bars: **A**, 30 μm, inset: 10 μm.

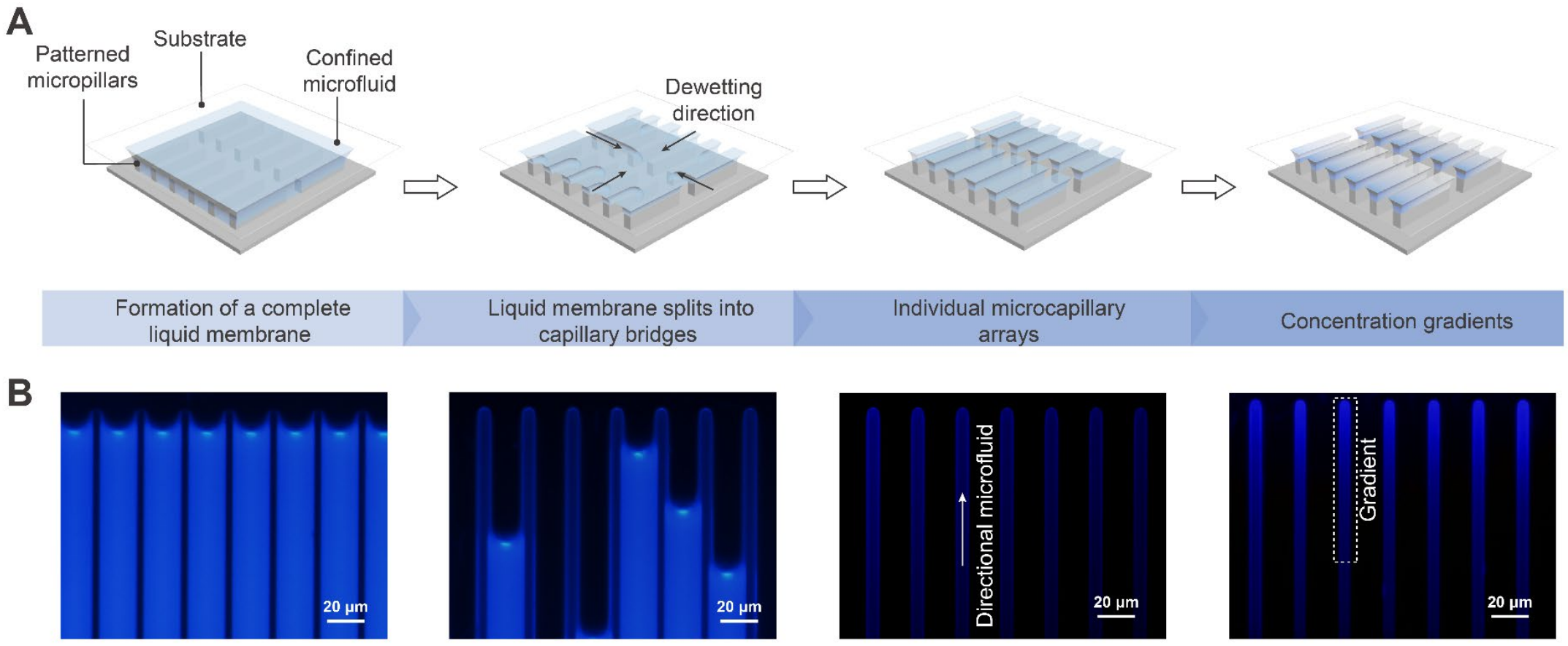

Fig. S2.

Illustration of dewetting process in gradient evaporation system. (**A**) Schemes of de-wetting progress and (**B**) the corresponding in-situ fluorescence microscopy images of microenvironment construction in gradient evaporation system, natural volatile condition. The apparent fluorescence intensity difference on the capillary bridges signified the formation of gradient concentration from the middle site to air end.

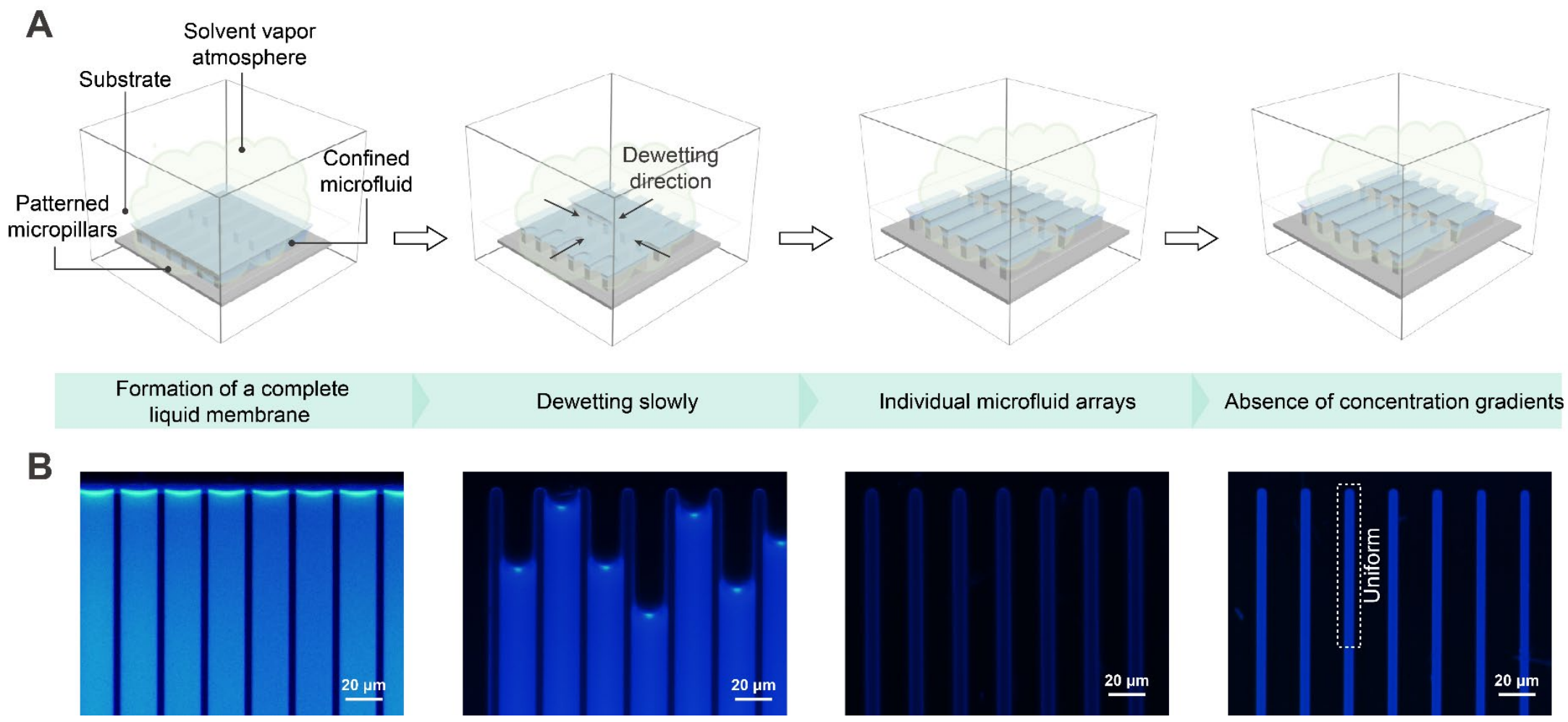

Fig. S3.

Illustration of dewetting process in confined evaporation system. (**A**) Schemes of de-wetting progress and (**B**) the corresponding in-situ fluorescence microscopy images of microenvironment construction in confined evaporation system, inhibited by solvent vapor atmosphere. Solvent evaporation on microcapillary bridges in confined evaporation system presented overall homogeneous states.

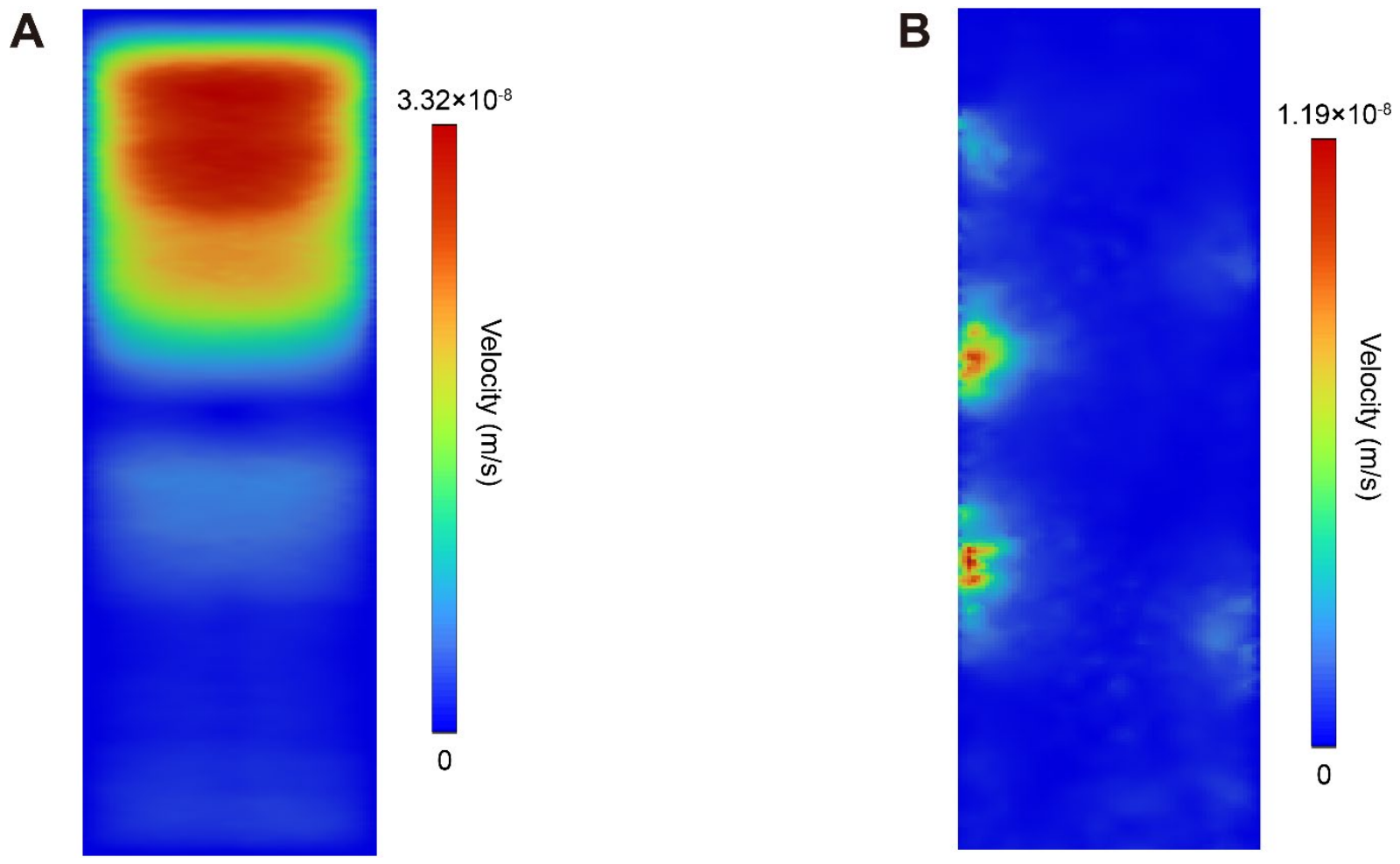

Fig. S4.
Computational fluid dynamics (CFD) simulations. Evaporation velocity distributions over microcapillary bridges in (**A**) gradient evaporation system and (**B**) confined evaporation system.

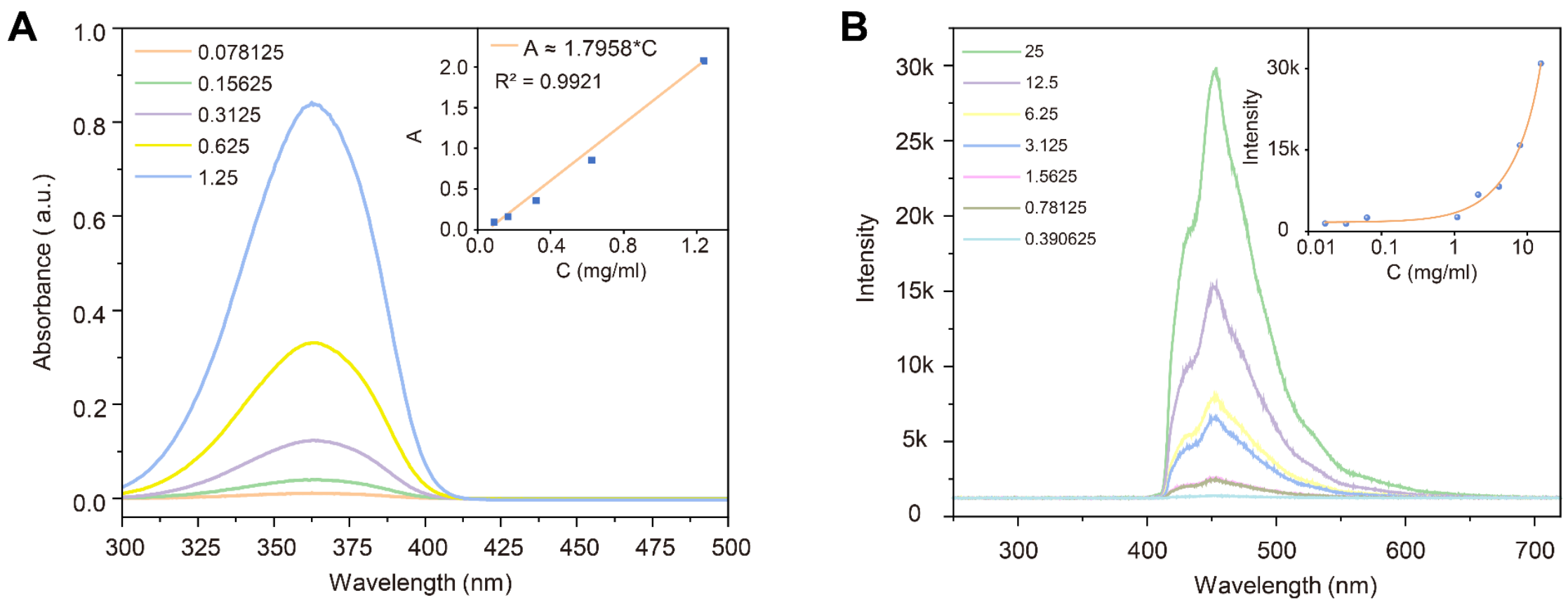

Fig. S5.

Saturation solubility characterization of polycrystalline organic DATP. (**A**) Absorption spectra of DATP with different concentrations in trichloromethane (chloroform) solvents, respectively. Inset, the dependence of DATP absorption on concentration at chloroform solvents through Lambert-Beer law. (**B**) The correlation between solution fluorescence emission intensity and solution concentration.

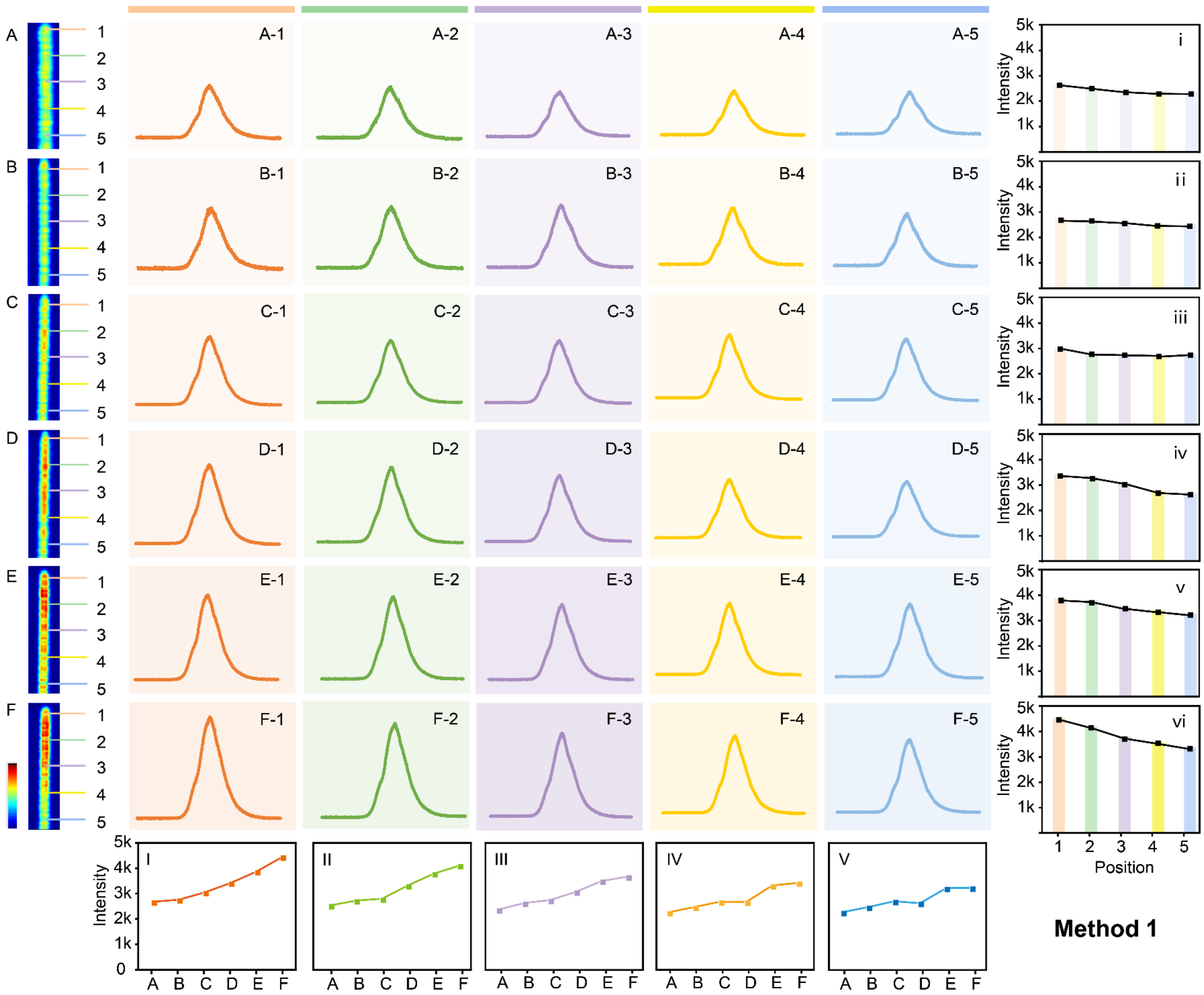

Fig. S6.

Fluorescence intensity mapping of gradient evaporation system during the dewetting process. (**A–F**) Six sequential fluorescence mapping images recorded to monitor the dewetting process. In each mapping image, five points at equal intervals along the gradient were selected, labeled 1–5, and their corresponding fluorescence emission spectra under 405 nm excitation are shown as A-1 to A-5, B-1 to B-5, and so on. The inset plots I–V compare the fluorescence emission intensity at the same position over time, whereas i–vi compare the fluorescence emission intensity at different positions at a given time. The spatial and temporal evolution of the fluorescence intensity confirms the successful construction of a gradient concentration microenvironment for nucleation.

A B C

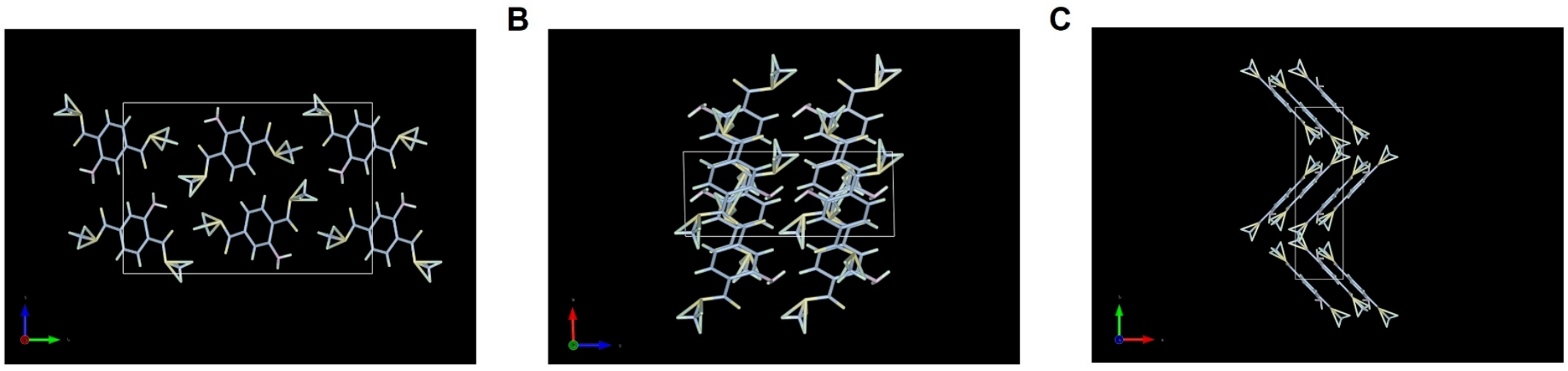

Fig. S7.

The unit cell structure of α phase of DATP. Single crystal packing of α phase view along a direction (**A**), view along b direction (**B**) and view along c direction (**C**).

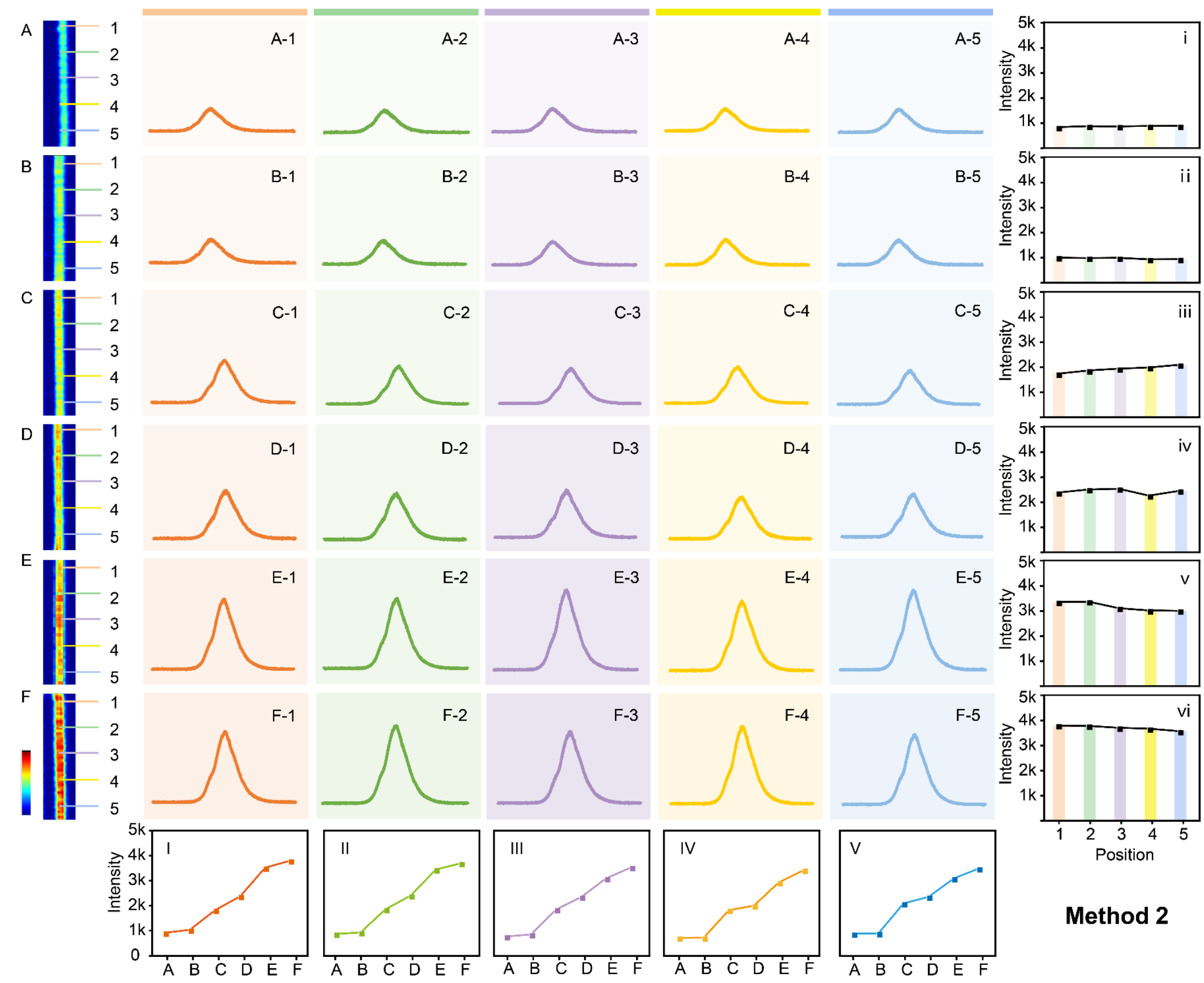

Fig. S8.

Fluorescence intensity mapping of confined evaporation system. (A–F) Six sequential fluorescence mapping images recorded during the confined evaporation process. In each image, five points at equal intervals were selected, labeled 1–5, and their corresponding fluorescence emission spectra under 405 nm excitation are shown as A-1 to A-5, B-1 to B-5, and so on. The inset plots I–V compare the fluorescence emission intensity at the same position over time, whereas i–vi compare the fluorescence emission intensity at different positions at a given time. The nearly uniform fluorescence intensity distribution and consistent evolution trends across different positions indicate homogeneous solvent evaporation in the confined evaporation system.

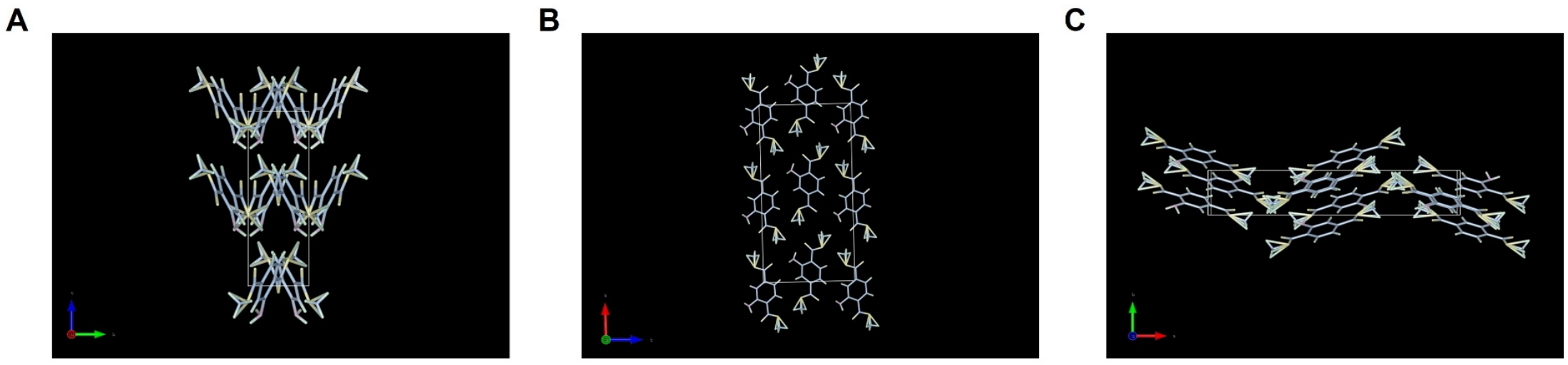

Fig. S9.

The unit cell structure of β phase of DATP. Single crystal packing of β phase view along a direction (**A**), view along b direction (**B**) and view along c direction (**C**).

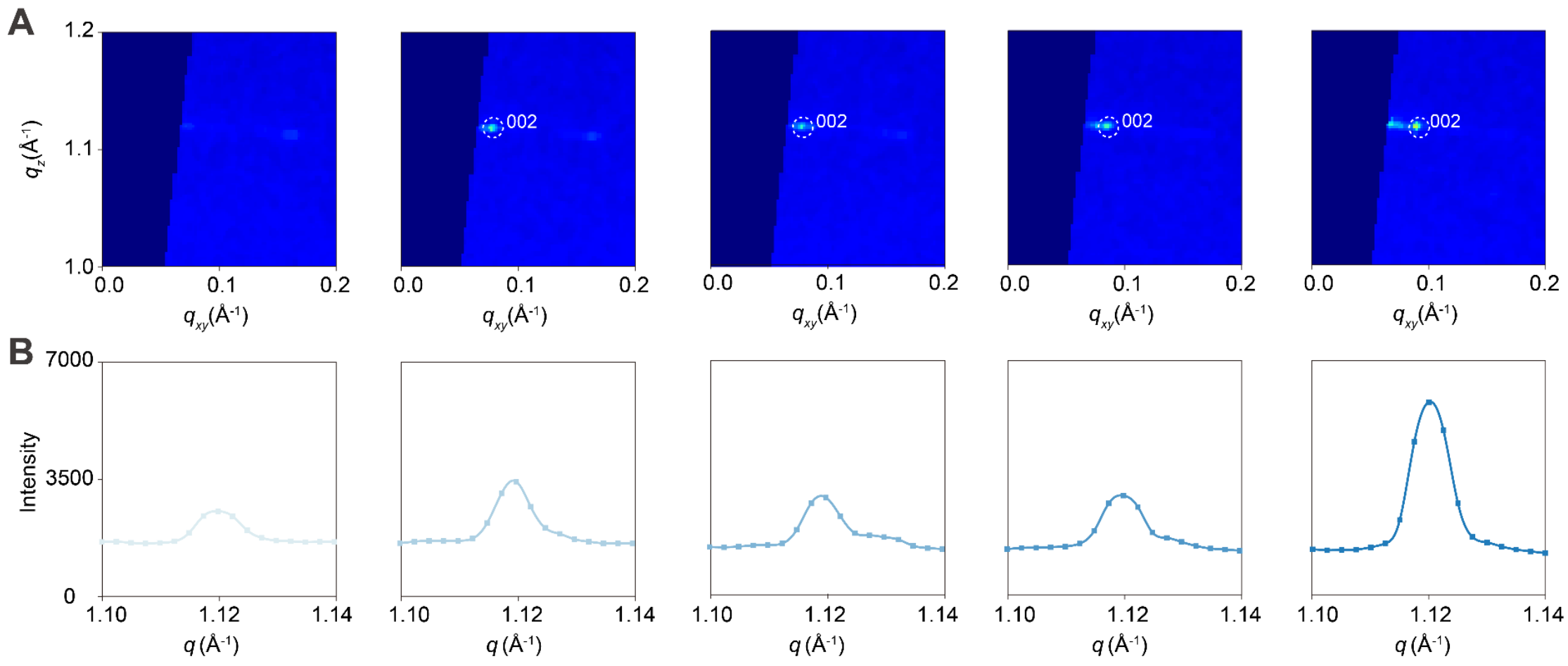

Fig. S10.

Time-resolved 2D GIWAXS patterns of α phase captured during nucleation. (A) Sequential 2D GIWAXS patterns recorded during the nucleation process, showing the appearance and progressive enhancement of the α-phase diffraction spot, indexed as the (002) reflection (dashed circles). **(B)** Corresponding intensity profiles in the selected q range, further revealing the emergence and gradual increase of the α-phase diffraction peak over time. These results indicate the nucleation and growth of the α phase.

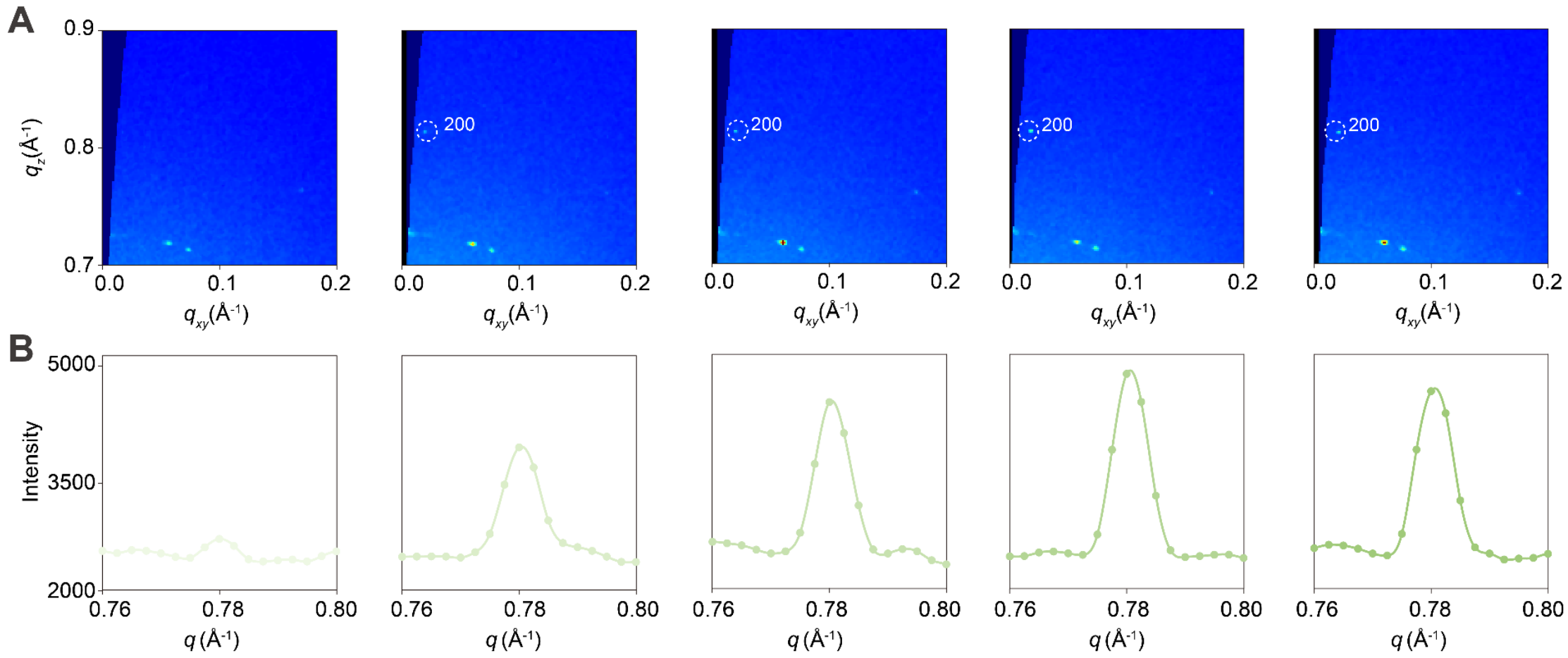

Fig. S11.

Time-resolved 2D GIWAXS patterns of *β* phase captured during nucleation. (A) Sequential 2D GIWAXS patterns recorded during the nucleation process, showing the appearance and progressive enhancement of the *β*-phase diffraction spot, indexed as the (200) reflection (dashed circles). **(B)** Corresponding intensity profiles in the selected *q* range, further demonstrating the emergence and gradual increase of the *β*-phase diffraction peak over time. These results confirm the nucleation and growth of the *β* phase.

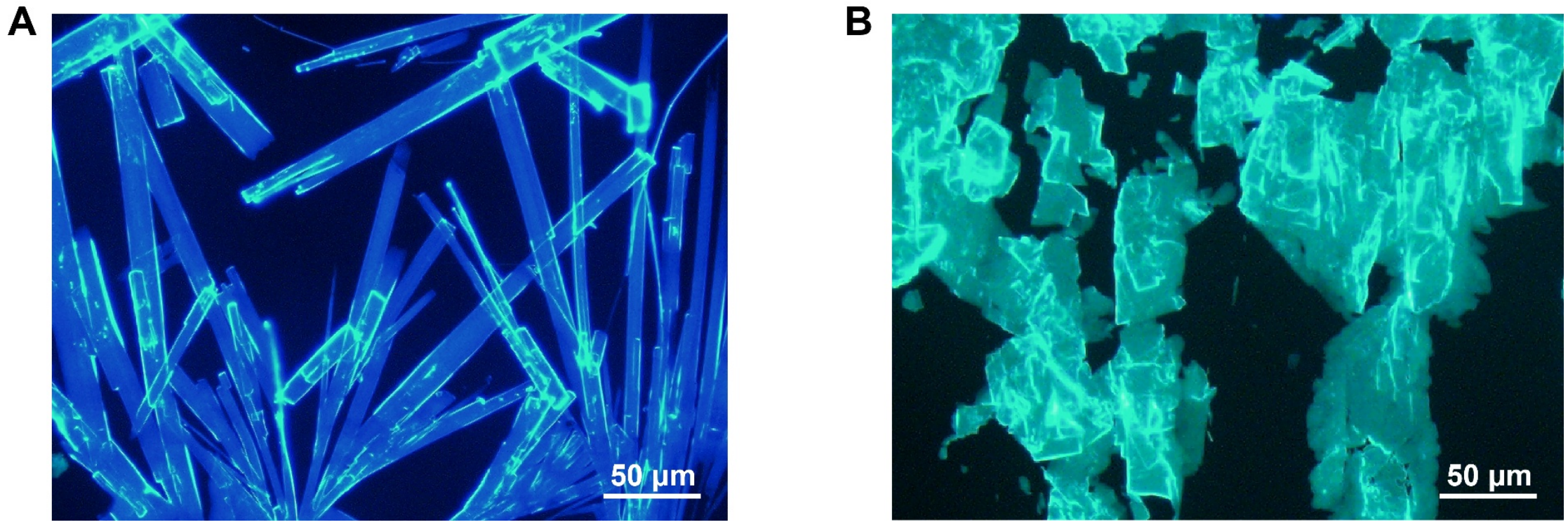

Fig. S12.

Morphological characterization of pristine DATP crystals without confined self-assembly patterning. Fluorescence microscopy (FM) images of DATP crystals in the (**A**) α phase and (**B**) β phase. The two crystalline phases exhibit distinct morphologies and fluorescence appearances in the absence of confined self-assembly patterning. Scale bars: 50 μm.

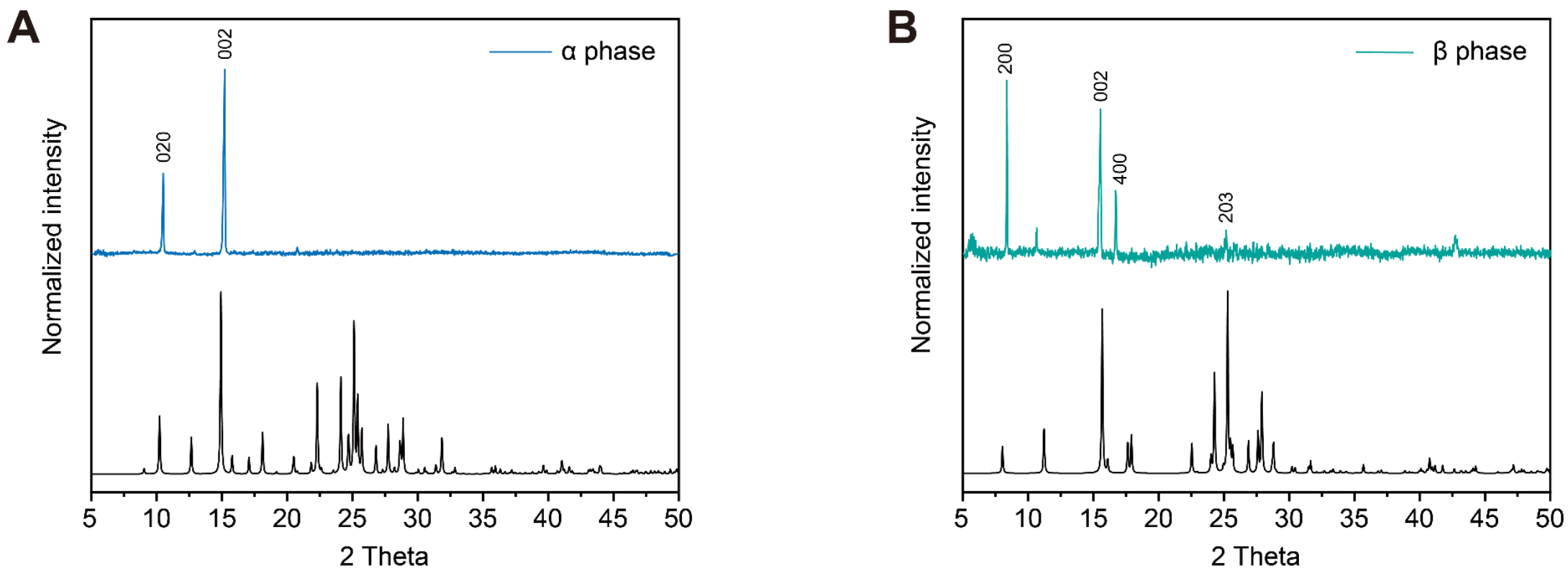

Fig. S13.

The XRD patterns of DATP. (**A**) α phase (blue line), (**B**) β phase (green line), and their standard crystal data (black line).

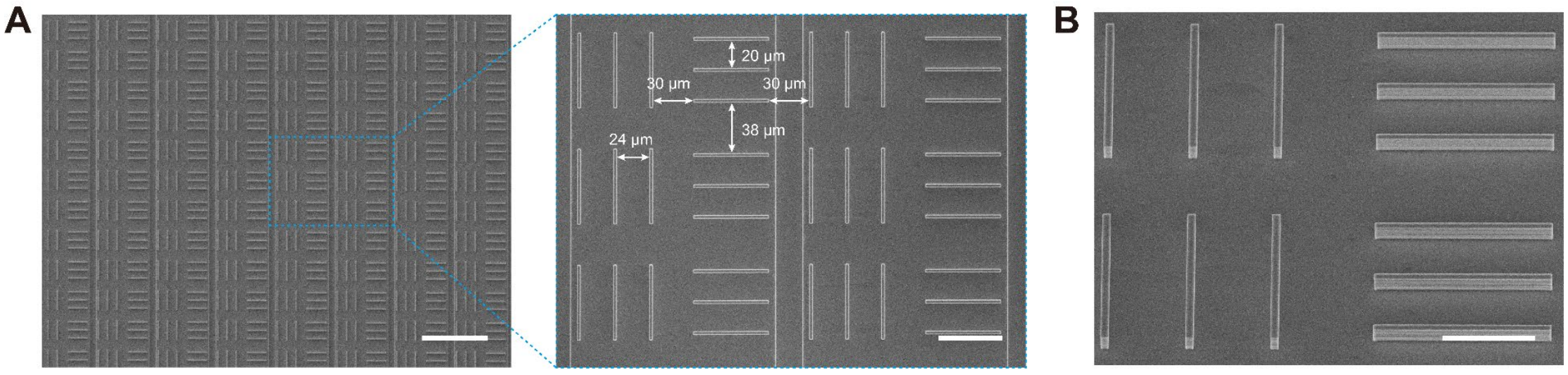

Fig. S14.
SEM of hierarchical silicon micropillar array platform. (**A**) Specific gap design enables spatial programming of multiple coexisting microenvironments within a single substrate. Zoom: specific parameters. (**B**) SEM image taken at 60° oblique angle provides a clearer view of the microcolumn array morphology. Scale bar: **A**, 200 µm, zoom in **A**: 50µm, **B**: 30 µm.

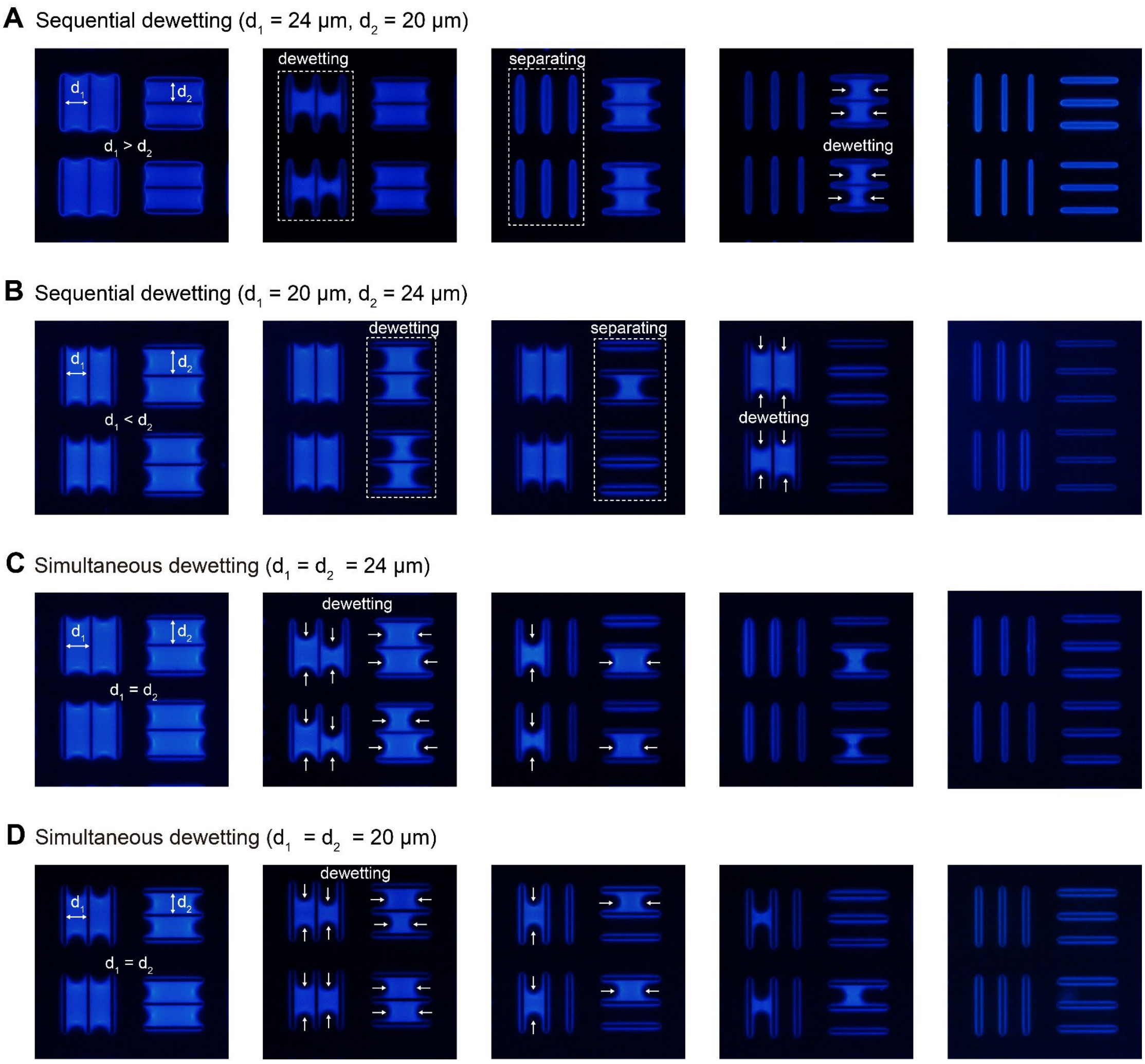

Fig. S15

In situ fluorescence microscopy images of dewetting dynamics in hierarchical silicon micropillar arrays with different width spacings. (**A**) Sequential dewetting in an array with d_1=24 μm and d_2=20 μm. The liquid film first dewets and separates in the region with the larger spacing, followed by dewetting in the smaller-spacing region. (**B**) Sequential dewetting in an array with d_1=20 μm and d_2=24 μm, again showing preferential dewetting in the wider-spacing region. (**C, D**) Simultaneous dewetting in arrays with equal spacings, d_1=d_2=24 μm and d_1=d_2=20 μm, respectively. Images in each row are arranged in chronological order from left to right. White dashed boxes highlight the regions where dewetting or filament separation occurs, and arrows indicate the receding liquid fronts.

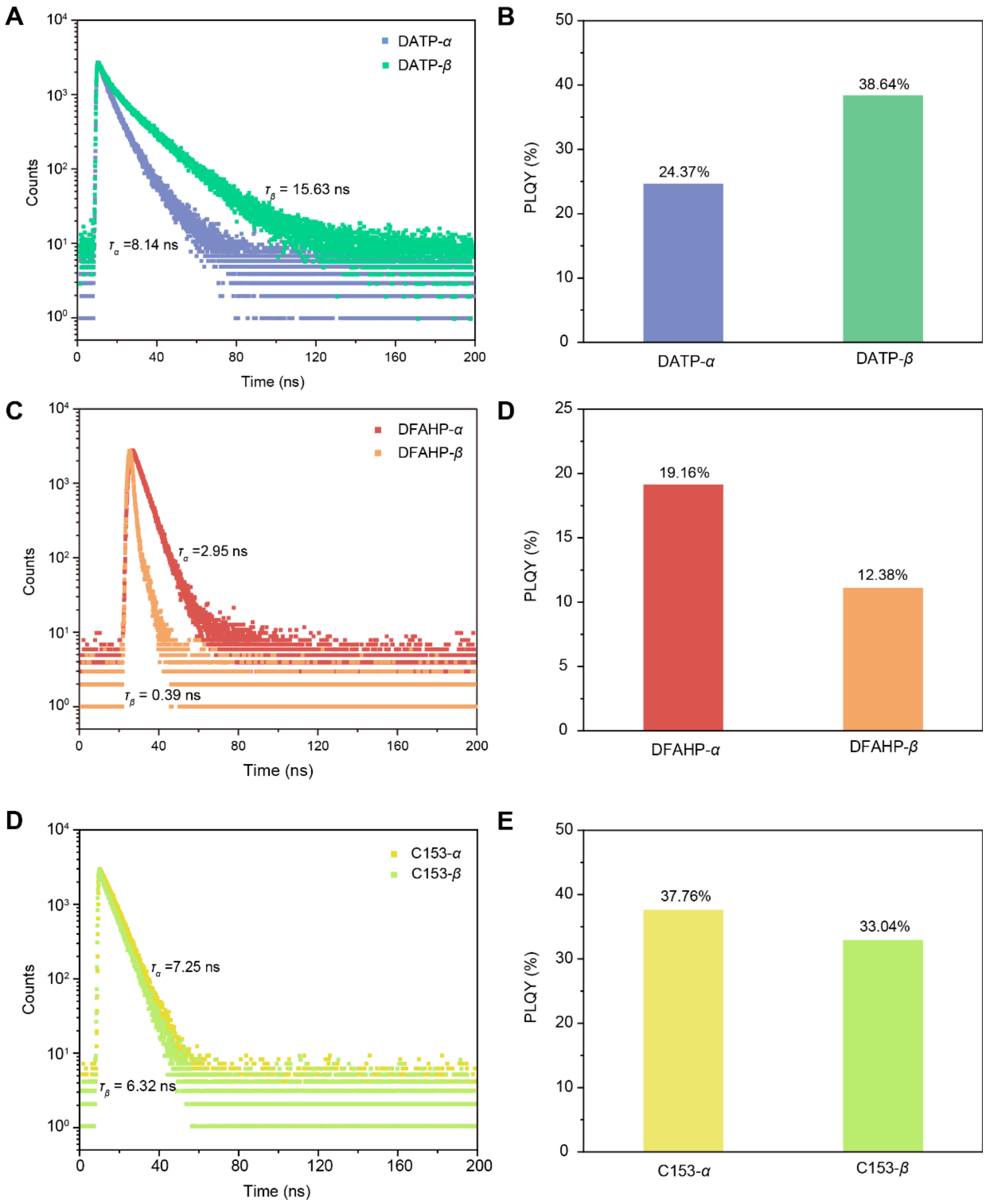

Fig. S16

Time-resolved photoluminescence (TRPL) decay and photoluminescence quantum yield (PLQY) of different polymorphic systems. Left panels (**A**, **C**, **D**): TRPL decay curves showing emission lifetimes of (**A**) DATP, (**C**) DFAHP and (**D**) C153. The extracted lifetimes (τ) indicate distinct excited-state dynamics between different polymorphs. Right panels (**B**, **D**, **E**): Corresponding PLQY values of each system, demonstrating the variation in radiative efficiency between α- and β-phases.

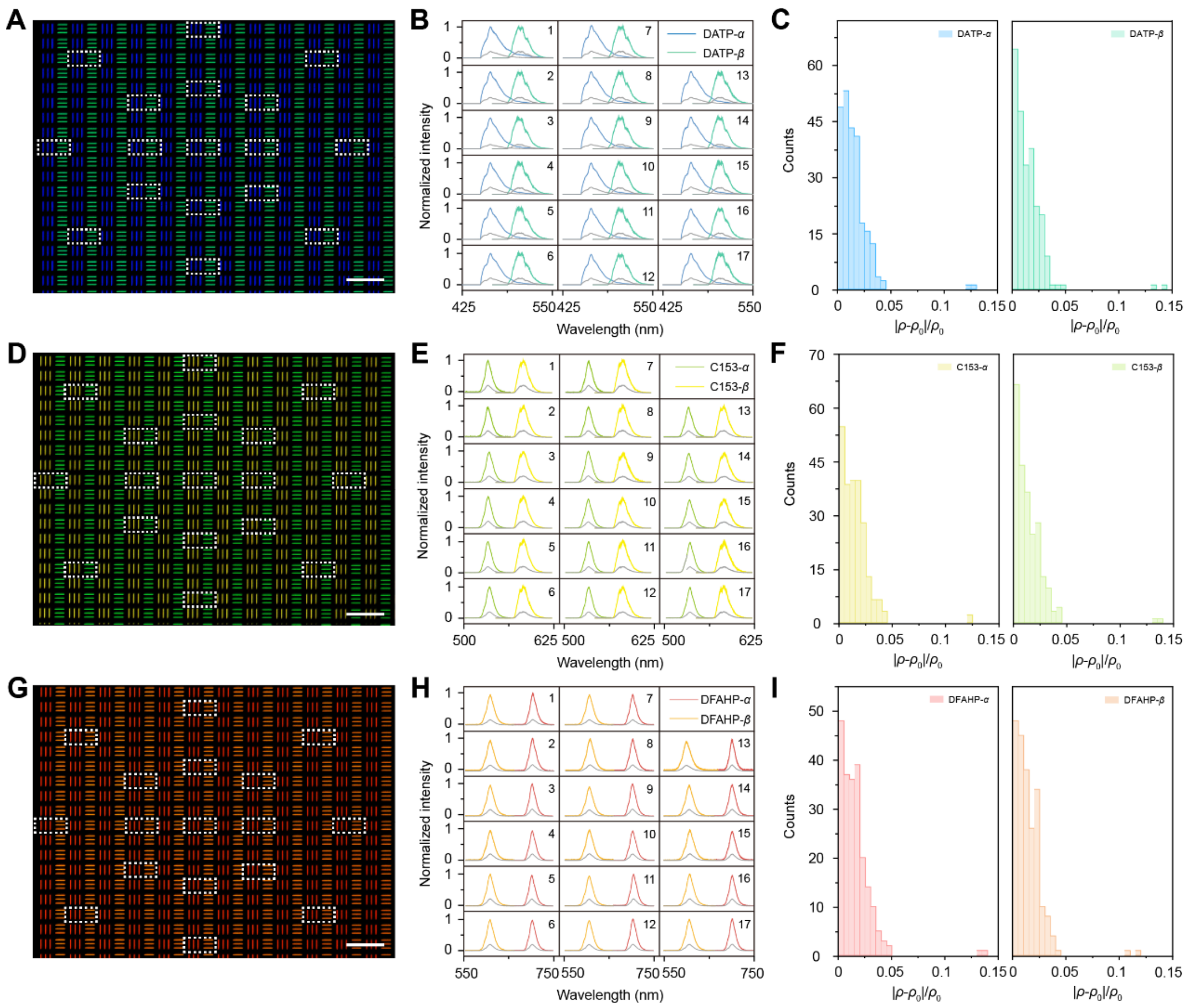

Fig. S17.

Large-area fluorescence uniformity and polarization-dependent emission of patterned arrays for three representative systems. (**A, D, G**) Wide-field fluorescence images of the patterned arrays under fixed excitation, showing highly uniform emission over large areas. The white dashed boxes indicate the sampling positions selected for local spectral analysis. (**B, E, H**) Normalized emission spectra collected from the corresponding sampling positions under analyzer angles of 0° and 90°, revealing pronounced polarization-dependent emission while maintaining good spectral consistency across different locations. (**C, F, I**) Histograms of the normalized spectral deviation, $|\rho\text{-}\rho_0|/\rho_0$, for the α- and β-phase populations of DATP, C153, and DFAHP, respectively. The polarization ratio is defined as $\rho = I_{\parallel} / I_{\perp}$. A reference polarization ratio ρ_0 was established from a well-characterized single-phase crystal. Crystals for which the relative deviation $|\rho\text{-}\rho_0|/\rho_0$ exceeded 10% were classified as impure-phase crystals. The narrow distributions centered near zero indicate high spectral uniformity across the arrays and reliable discrimination between the two polymorphic optical channels. Scale bars, 250 μm.

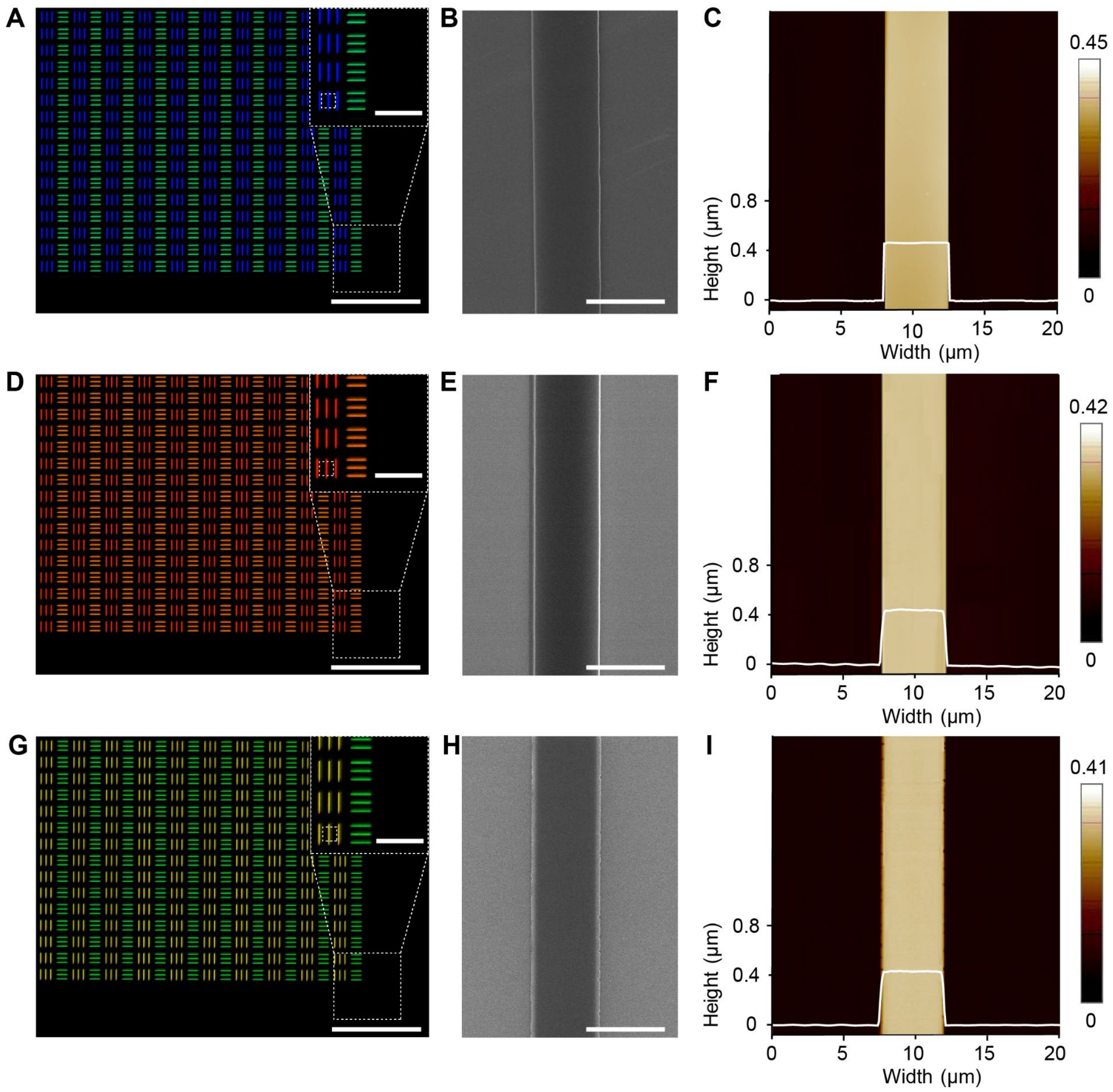

Fig. S18.

Processing quality and structural uniformity of polymorphic fluorescent microarrays. (**A**, **D**, **G**) Large-area fluorescence image of the patterned polymorphic microarray with clear pattern boundaries, regular spatial arrangement, and uniform fluorescence over the patterned area; the enlarged insets further highlight the well-defined local features. (**B**, **E**, **H**) Enlarged scanning electron microscope (SEM) image of an individual patterned unit, showing a straight-edged and well-retained rectangular microstructure. (**C**, **F**, **I**) Atomic force microscopy (AFM) image showing the topographic height map and corresponding line-scan profile revealing flat top surface. Scale bars: (**A**, **D**, **G**) 500 μm, inset: 150 μm, (**B**, **E**, **H**) 5 μm.

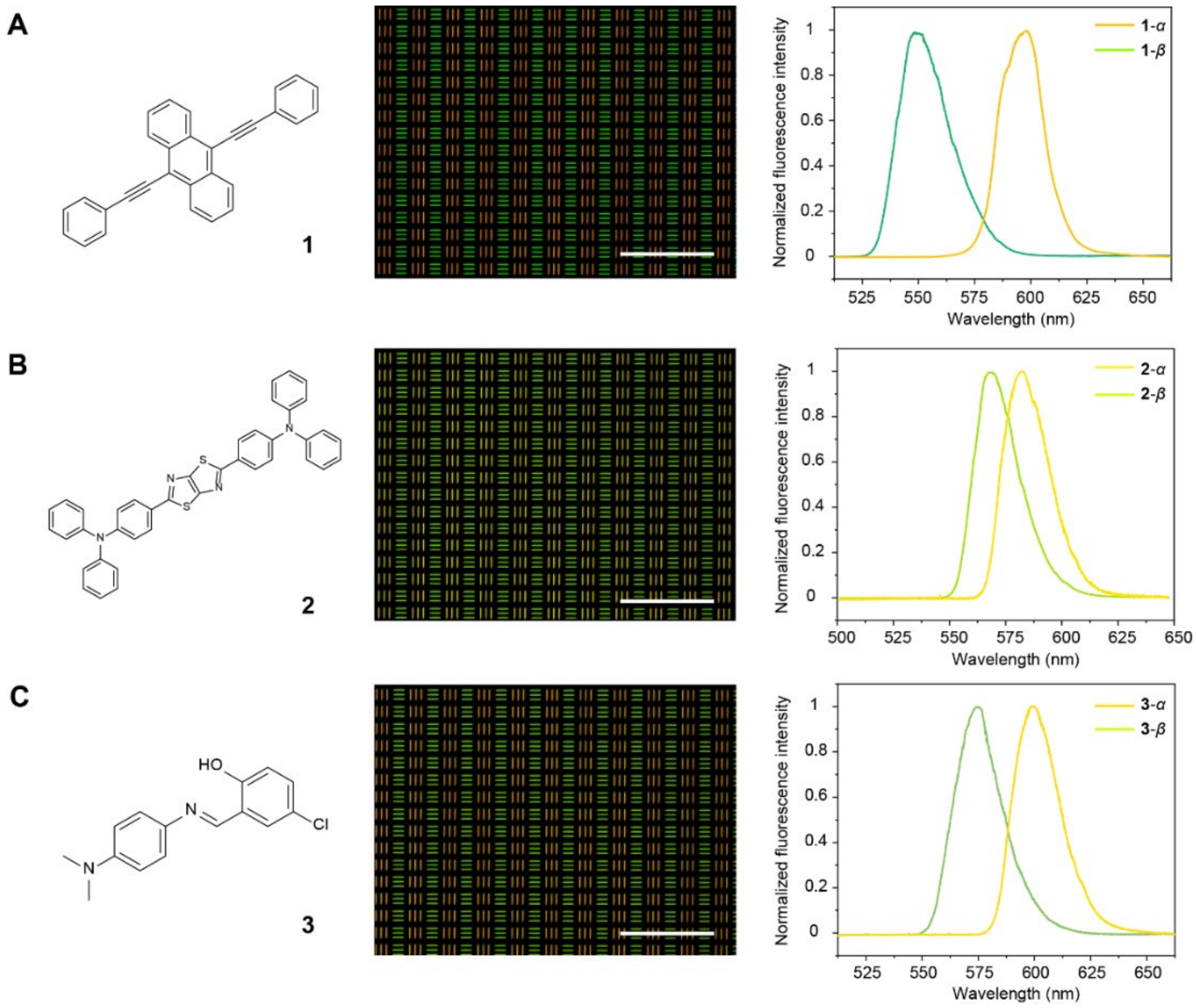

Fig. S19.

Universality of the topological template-assisted polymorphic crystallization strategy for different fluorescent molecular systems. (**A**, **B**, **C**) Representative fluorescence images of ordered polymorphic microarrays fabricated from three distinct organic fluorescent systems under identical topological confinement and microfluidic-guided crystallization conditions. In all cases, highly ordered array structures with uniform spatial arrangement and controlled crystal orientation were successfully obtained over large areas. Molecule **1**: 9,10-bis(phenylethynyl)anthracene; Molecule **2**: (4,4′-(thiazolo[5,4-*d*]-thiazole-2,5-diyl)bis(*N*,*N*-diphenylaniline); Molecule **3**: (*E*)-4-chloro-2-(((4-(dimethylamino)phenyl)imino)methyl)phenol. Scale bar: 500 μm.

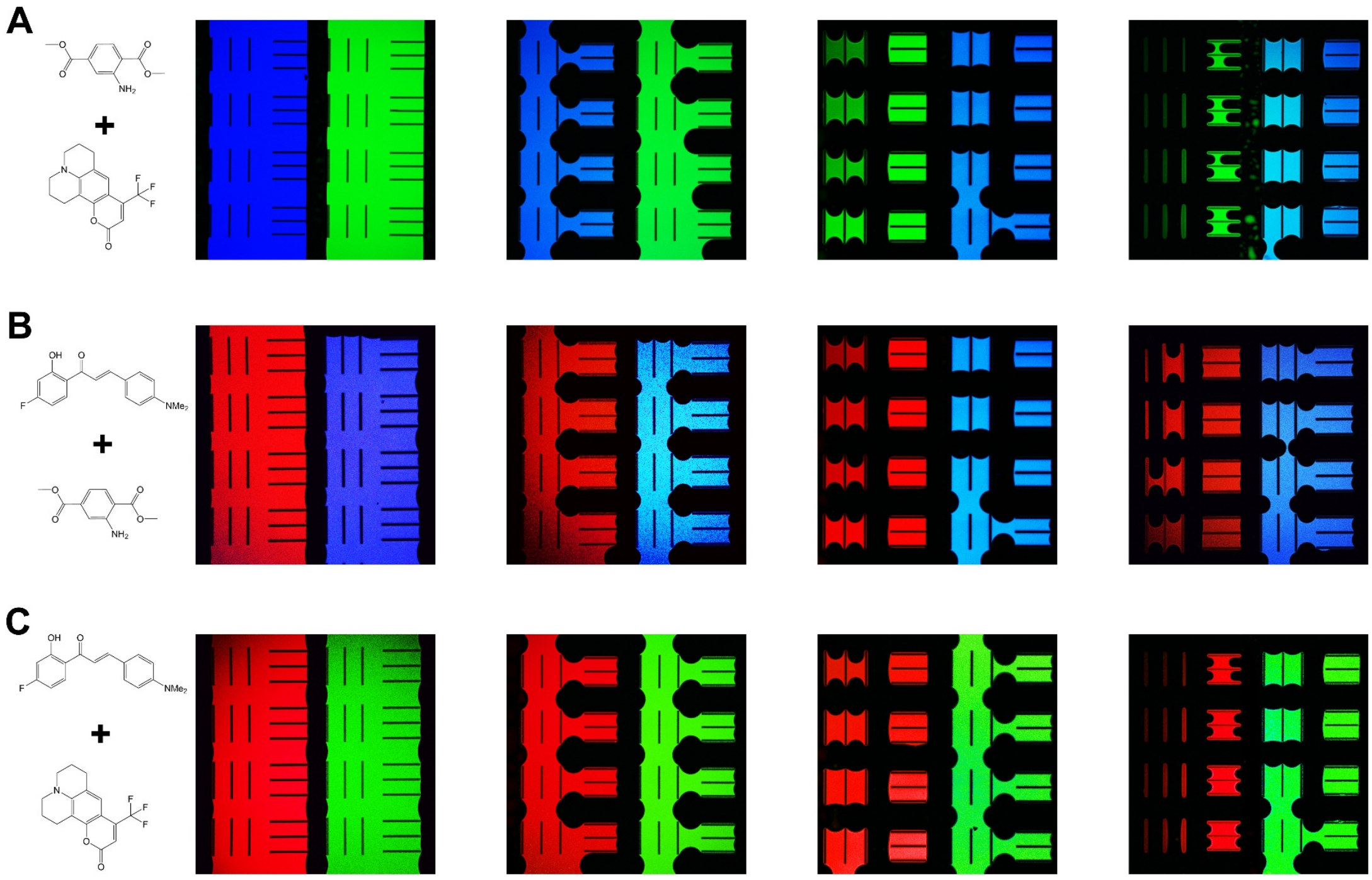

Fig. S20.

Confocal images of sequential dewetting in hierarchical silicon micropillar array platform. (**A**) DATP and C153; (**B**) DFAHP and DATP; (**C**) DFAHP and C153, illustrating the evolution of liquid redistribution and the formation of spatially separated fluorescent microdomains during dewetting. The fluorescence signals are displayed in pseudo-colors corresponding to DATP (blue), C153 (green), and DFAHP (red).

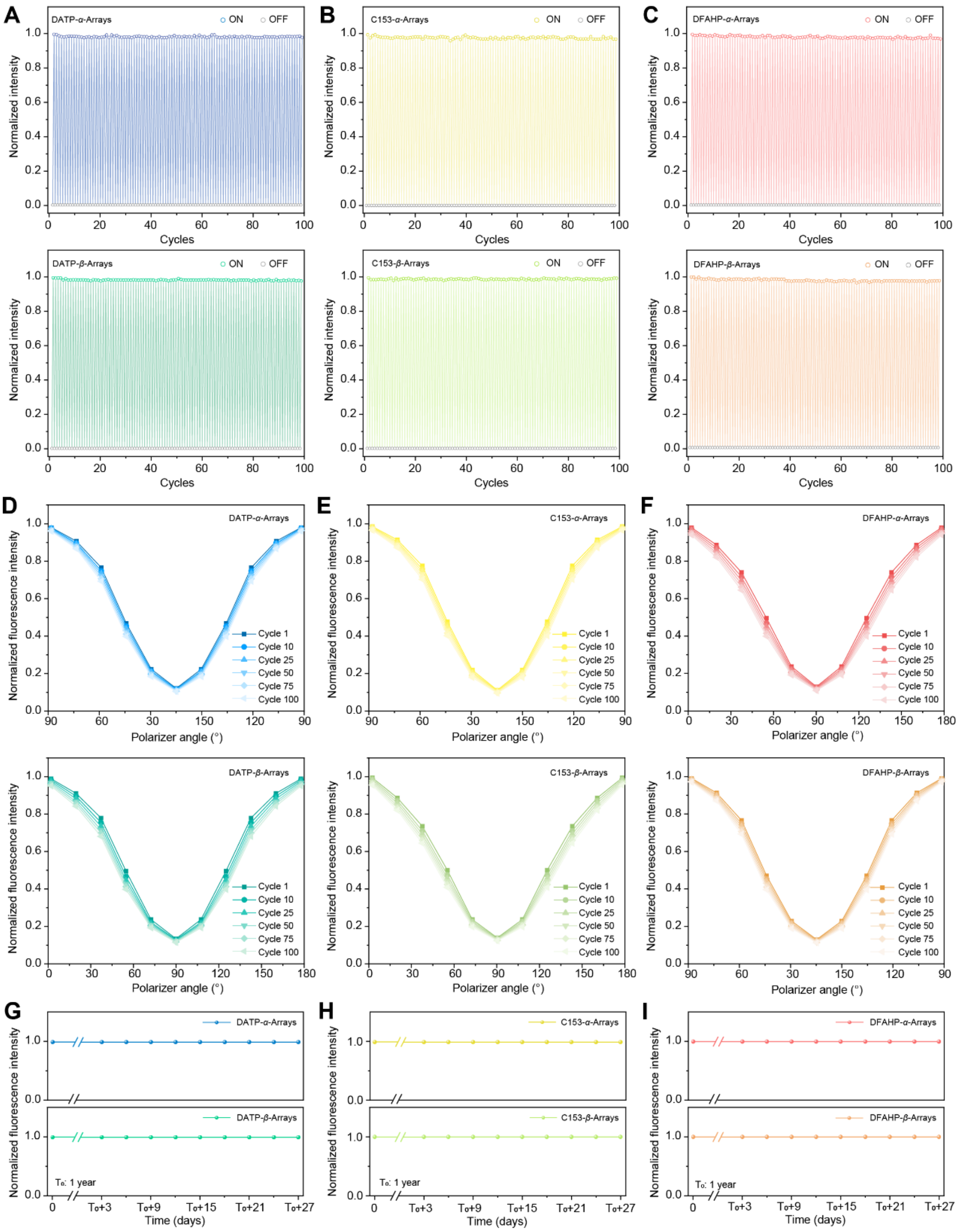

Fig. S21.

Reversible switching durability, polarization retention, and storage stability of DATP, C153, and DFAHP arrays. (**A**, **B**, **C**) Normalized fluorescence intensities of the *α*/*β* arrays of DATP, C153, and DFAHP during 100 consecutive switching cycles. Colored circles represent the "ON" state, while gray circles represent the "OFF" state. All arrays exhibit stable fluorescence outputs and high ON/OFF contrast throughout repeated cycling, indicating excellent reversibility and fatigue resistance. (**D**, **E**, **F**) Polarization-dependent fluorescence intensity profiles of the *α*/*β* arrays of DATP,

C153, and DFAHP recorded after different cycle numbers (Cycle 1, 10, 25, 50, 75, and 100). All samples retain pronounced angle-dependent emission behavior after repeated switching, with nearly unchanged curve shapes and only slight intensity attenuation, demonstrating robust polarization stability upon cycling. (**G**, **H**, **I**) Time-dependent normalized fluorescence intensities of the *α*/*β* arrays of DATP, C153, and DFAHP over one year and 27 days of storage. T_0 indicates that the test sample had been stored for one year before testing. The fluorescence intensities remain nearly unchanged over time, confirming excellent long-term storage stability.

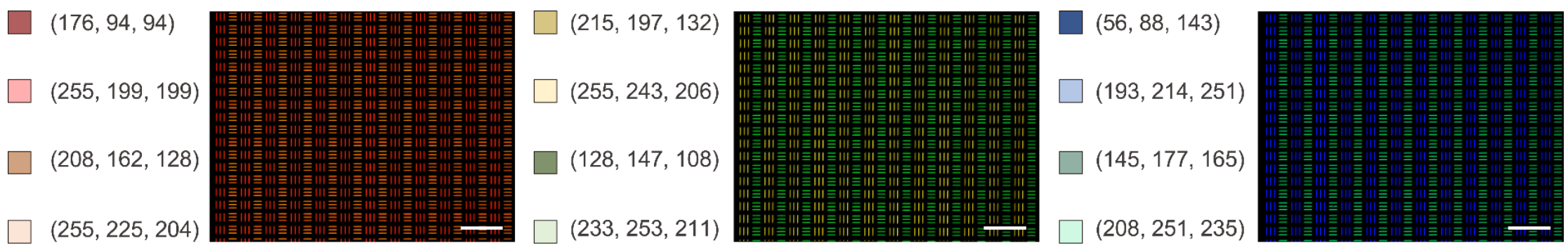

Fig. S22

Correlation between optical information and pseudo-color RGB values.

Representative pseudo-color renderings of the encoded microarrays using different RGB assignments. For each optical channel, four representative RGB tuples are listed alongside the corresponding reconstructed array image, demonstrating the direct correspondence between the stored optical information and the assigned pseudo-color parameters. Scale bars, 300 µm.

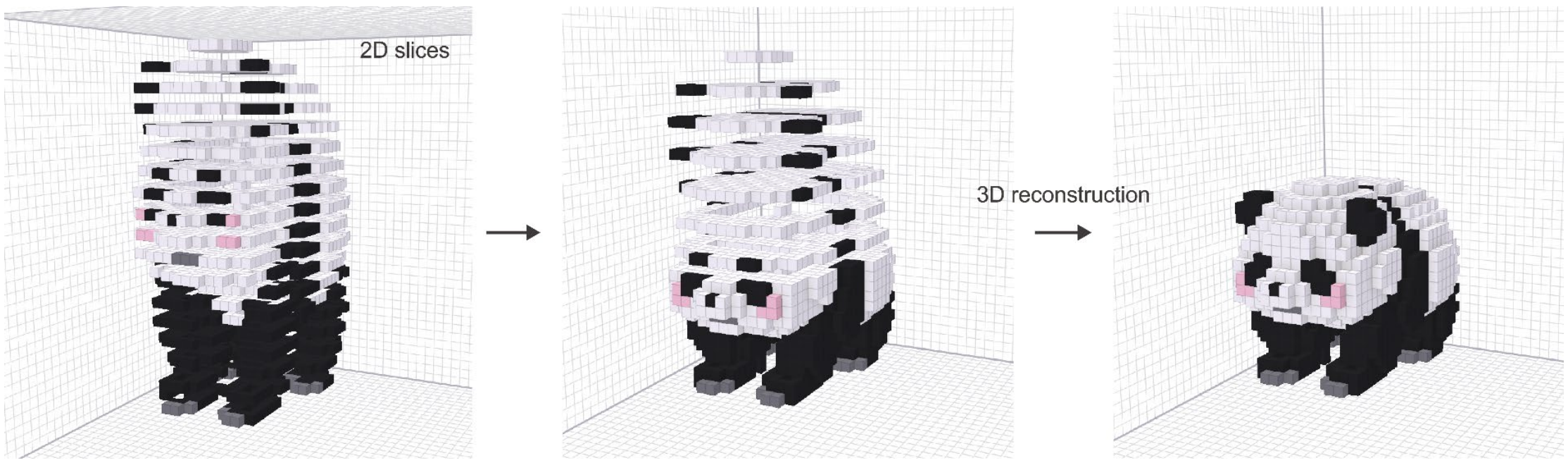

Fig. S23.

3D extension of multi-dimensional optical encryption. Correlation between optical information and pseudo-color RGB values and schematic illustration of the 3D model reconstruction process. The encoded optical information is first converted into a series of pseudo-colored 2D slices according to the assigned RGB values, which are then sequentially stacked to generate the final 3D voxel-based model. The panels illustrate the workflow from 2D slice acquisition, to intermediate slice assembly, to the reconstructed 3D object.

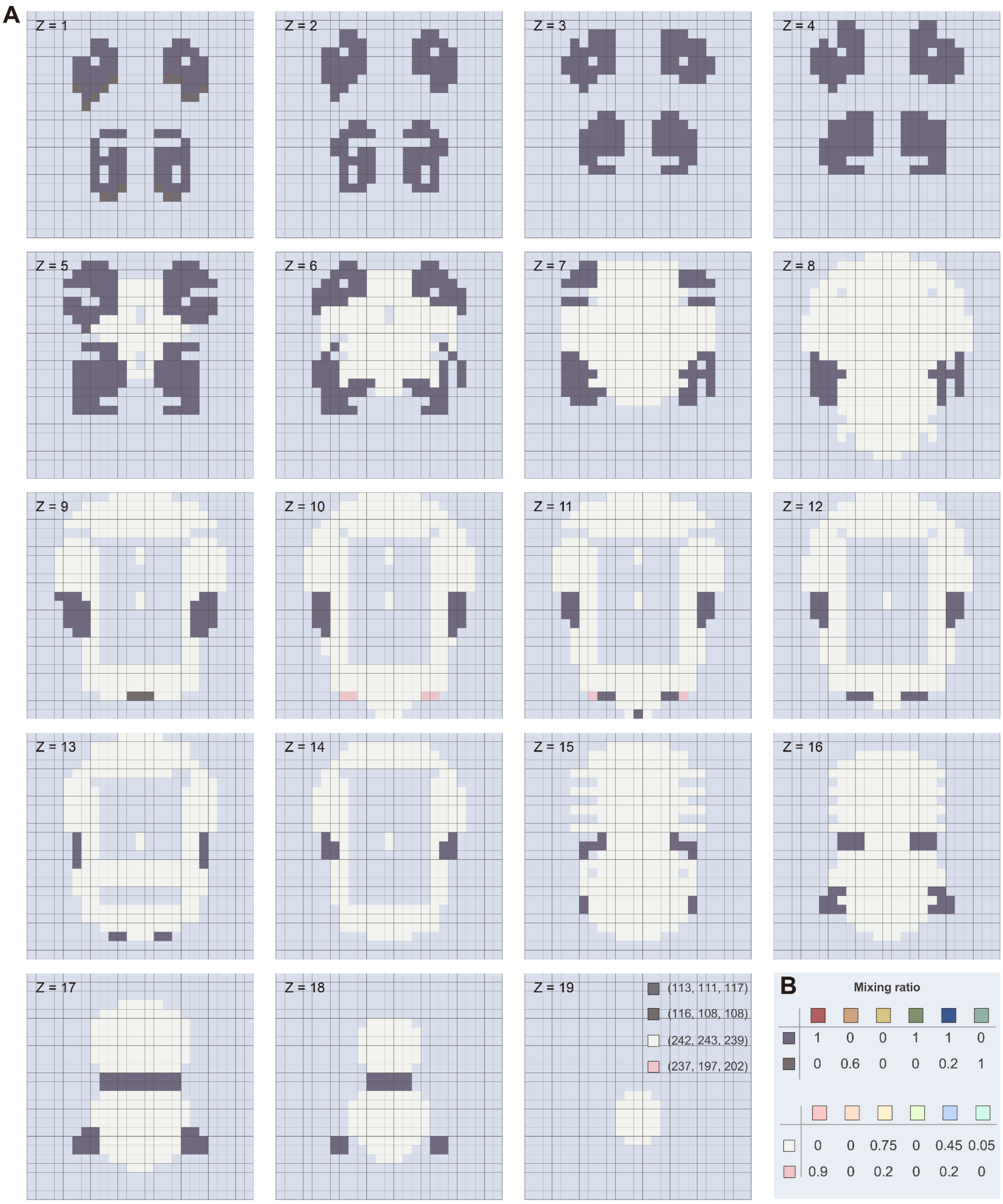

Fig. S24.

Reconstruction process of the 3D panda model. (**A**) Encode/decode outputs of the individual single-layer slices used to reconstruct the 3D panda model, shown sequentially from Z=1 to Z=19. Each slice represents the pixelated optical information at a specific height of the 3D structure. (**B**) RGB-weighted mixing algorithms used for color generation in the reconstruction process. Different target pseudo-colors are produced by applying predefined weighting ratios to the RGB channels, enabling accurate color assignment for each encoded slice and subsequent 3D model reconstruction.

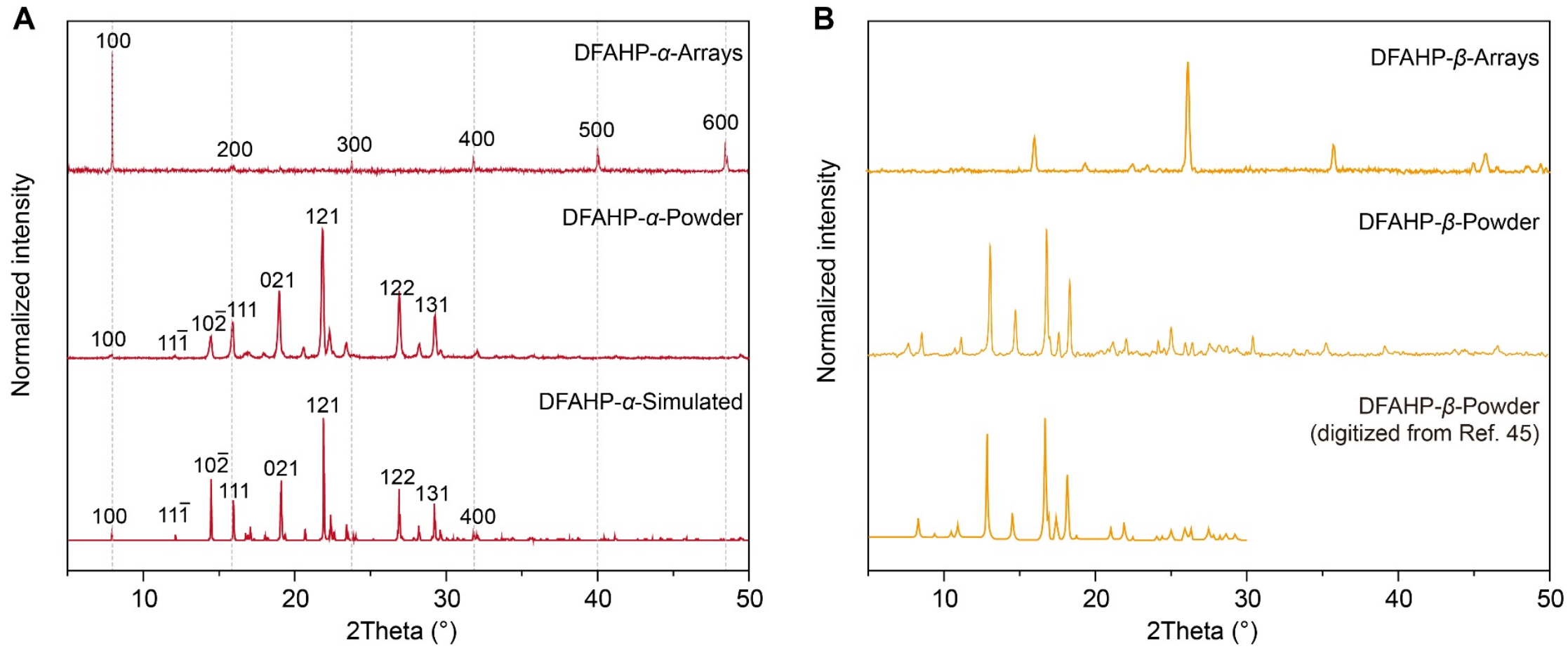

Fig. S25.

Powder X-ray diffraction (PXRD) patterns of DFAHP polymorphs. (**A**) Comparison of experimental PXRD patterns for DFAHP-*α* arrays and DFAHP-*α* powder with the simulated pattern of the DFAHP-*α* phase. The diffraction peaks of the array sample are consistent with simulated patterns, confirming the successful formation of the targeted polymorph in the patterned structures. (**B**) PXRD patterns of DFAHP-*β* arrays and DFAHP-*β* powder, compared with PXRD pattern of DFAHP-*β* from Ref 45. The *β*-phase maintains good agreement between array/powder samples and PXRD pattern of DFAHP-*β* from Ref 45, supporting the assignment of the right polymorph.
